# Roles of logistics service quality in shaping generation Z customers’ repurchase intention and electronic word of mouth in E-commerce industry

**DOI:** 10.1371/journal.pone.0323962

**Published:** 2025-05-28

**Authors:** Thi Thuy An Ngo, Gia Khuong An, Dang Khoa Dao, Ngoc Quynh Nhu Nguyen, Ngoc Yen Vy Nguyen, Bao Han Phong

**Affiliations:** 1 Department of Business Administration, FPT University, Can Tho City, Vietnam; 2 Department of International Business, FPT University, Can Tho City, Vietnam; Al-Ahliyya Amman University, JORDAN

## Abstract

As e-commerce continues to reshape retail landscapes, logistics service quality (LSQ) has become a crucial determinant of customer trust, satisfaction, and long-term engagement. This study investigates the impact of logistics service quality (LSQ) dimensions on the behavioral intentions of Generation Z consumers within Vietnam’s rapidly expanding e-commerce sector. The research focuses on how various LSQ factors—timeliness, personal contact quality, order accuracy, order condition, order discrepancy handling, and return convenience—affect trust and satisfaction, which subsequently influence repurchase intention and electronic word-of-mouth (eWOM). A quantitative approach was employed, gathering data from 495 Generation Z consumers with prior online shopping experience. Partial Least Squares Structural Equation Modeling (PLS-SEM) was used to test the proposed model and its hypotheses. This study found that key LSQ attributes play a significant role in shaping both trust and satisfaction, which, in turn, drive repurchase intention and eWOM. However, the findings indicate that Generation Z’s expectations for seamless logistics experiences vary across different service attributes. While factors such as order accuracy, order condition, and order discrepancy handling contribute to satisfaction, they do not necessarily build trust, highlighting the generation’s high standards and perception of these aspects as fundamental rather than differentiating features. This study challenges traditional LSQ frameworks by highlighting the evolving expectations of digital-native consumers. It offers practical insights for e-commerce businesses, emphasizing the need for a strategic blend of technological efficiency, personalized interactions, and seamless post-purchase services to enhance customer loyalty and competitiveness in the digital marketplace.

## 1. Introduction

The rapid acceleration of digital transformation has fundamentally reshaped the global retail landscape, positioning e-commerce as a dominant force in consumer markets. Among the various demographic groups driving this shift, Generation Z stands out for its profound impact on online shopping trends, characterized by its digital fluency, sophisticated consumption patterns, and elevated service expectations [1]. This generational transformation is not confined to any single market but represents a global phenomenon, shaping e-commerce trends across diverse regions, including North America, Europe, and emerging economies such as Vietnam. As one of Southeast Asia’s fastest-growing e-commerce markets, Vietnam exemplifies the dynamic interplay between digital adoption and consumer behavior, with Generation Z playing a crucial role in driving online commerce expansion [2,3]. Despite the growing influence of this demographic, existing theoretical frameworks often fail to adequately capture the generational and regional nuances that define their consumer behavior, highlighting the need for a more comprehensive understanding of their expectations and purchasing patterns [4]. This highlights a critical gap in understanding how traditional logistics service quality (LSQ) frameworks, largely developed before the rise of this digital-native generation, apply to their unique expectations and purchasing patterns, particularly in emerging markets. Furthermore, the interplay between cultural contexts, technological advancement levels, and LSQ perceptions in emerging markets represents an understudied area that requires urgent scholarly attention.

One of the critical aspects of this transformation is the increasing importance of logistics service quality (LSQ) in maintaining competitive advantage, fostering consumer trust, and ensuring customer retention in an increasingly competitive digital marketplace [5]. LSQ has been widely recognized as a critical determinant of e-commerce success, as highlighted by extensive research in the field [6]. The current literature presents several critical theoretical and practical gaps that this research aims to address. These include the inability of existing LSQ frameworks fail to capture the unique technological expectations and service preferences of Generation Z consumers, the limited understanding of how cultural and economic contexts in emerging markets shape LSQ perceptions and outcomes, the lack of integration between modern technological capabilities and traditional service quality dimensions, and the insufficient exploration of the complex mediating mechanisms linking LSQ dimensions to customer behavioral outcomes in emerging market contexts. Traditional LSQ models primarily focus on generalized metrics such as timeliness, order accuracy, and return convenience, often overlooking how these dimensions interact with generational and regional factors. In particular, younger consumers, especially Generation Z, may prioritize different aspects of service quality, such as delivery speed, seamless return processes, and digital integration, which are frequently underrepresented in conventional LSQ frameworks [7,8].

Despite the extensive body of research on LSQ, significant gaps remain in understanding how it shapes consumer behavior among Generation Z, particularly in emerging markets. For instance, Do et al. [5] emphasized that accurate order fulfillment, timely delivery, and convenient return processes play a pivotal role in retaining e-commerce customers. Similarly, Rashid and Rasheed [7] demonstrated that logistics dimensions such as product quality, delivery time, and the condition of goods not only enhance customer satisfaction but also promote positive electronic word-of-mouth (eWOM). These findings underscore LSQ’s integral role in shaping consumer loyalty and advocacy. However, most existing LSQ studies have been conducted in generalized or mature markets [9,10], often overlooking the unique characteristics of emerging markets and younger consumer cohorts. Generation Z, for example, exhibits a strong preference for technology-driven solutions and seamless experiences, which traditional LSQ frameworks may not fully address. Moreover, these frameworks typically overlook regional factors that could shape the perception of logistics quality in diverse cultural and economic contexts. Given that Generation Z consumers in Vietnam may have distinct expectations compared to those in developed economies [11], it is essential to reevaluate LSQ dimensions to ensure they align with the evolving behaviors and preferences of this demographic.

In the context of Vietnam, the exploration of LSQ takes on added significance. As one of Southeast Asia’s fastest-growing e-commerce markets, Vietnam represents a dynamic landscape characterized by rapid digital transformation and unique cultural influences. Vu et al. [12] noted that although the country has made remarkable economic strides, research on LSQ within its specific cultural and economic framework remains sparse. This limited attention has led to a fragmented understanding of how logistics services impact consumer trust, satisfaction, and loyalty in Vietnam’s burgeoning e-commerce ecosystem. Furthermore, while emerging markets worldwide share similar trends of rapid digital adoption and young, tech-savvy consumer bases, many LSQ models remain rooted in traditional service frameworks that do not adequately reflect these evolving market dynamics [13]. Addressing these gaps is critical as Vietnam’s e-commerce sector continues to expand, driven by increasing internet penetration and a growing Generation Z consumer base.

Current research on LSQ often adopts a segmented approach, examining individual dimensions such as timeliness, order accuracy, and return convenience [14,15]. However, these studies fail to offer an integrated perspective that captures the interplay of these dimensions and their cumulative impact on consumer behaviors, particularly repurchase intention and eWOM among Generation Z. This lack of a holistic framework presents a significant theoretical void, especially considering the nuanced role of mediators like trust and satisfaction in these relationships. Moreover, the absence of generational and regional insights in current LSQ models limits their applicability across diverse e-commerce markets. A more comprehensive and flexible LSQ framework is needed—one that accounts for the unique expectations of Generation Z consumers and the contextual factors shaping logistics perceptions in emerging markets. By addressing these limitations, research can contribute to a more nuanced understanding of LSQ’s role in fostering consumer trust, satisfaction, and long-term engagement in the evolving digital marketplace.

To address these gaps, this research proposes a comprehensive theoretical framework that examines the multifaceted relationships between LSQ dimensions and Generation Z’s behaviors in Vietnam’s e-commerce sector. The study addresses some specific objectives: Identify the most influential LSQ dimensions for Generation Z consumers in emerging markets, examine the mediating mechanisms through which LSQ affects customer outcomes, and develop a culturally contextualized framework for understanding e-commerce logistics in developing economies. Specifically, the study focuses on how LSQ dimensions—including timeliness, personal contact quality, order accuracy, order condition, discrepancy handling, and return convenience—interact to shape Generation Z’s repurchase intentions and eWOM behaviors. It further explores the mediating roles of trust and satisfaction in these relationships, offering a nuanced understanding of the mechanisms driving customer loyalty and advocacy. The theoretical foundation of this study builds on the frameworks of Akıl and Ungan [16], Do et al. [17], and Zia et al. [18], integrating their insights into a novel and comprehensive analytical perspective. By addressing these critical gaps, the research not only advances the understanding of LSQ in an emerging market context but also offers practical insights for e-commerce stakeholders aiming to cater to the unique needs of Generation Z consumers.

This research offers significant contributions to theory and practice. Theoretically, it advances LSQ literature by developing an integrated framework that accounts for generational preferences, cultural nuances, and technological advancement levels, factors that existing models have largely treated in isolation. By addressing generational and regional nuances, the framework offers insights that are not only relevant to Vietnam but also transferable across global markets, thus enhancing the understanding of how Generation Z’s distinct preferences influence e-commerce dynamics worldwide. Methodologically, it employs a rigorous approach that captures the complexity of modern logistics systems and their impact on customer behavior. This study employs a comprehensive structural equation modeling approach that simultaneously examines direct and indirect effects of LSQ dimensions, offering a more sophisticated analytical framework than previous single-dimension or linear analyses. Practically, this research addresses crucial challenges faced by e-commerce businesses in emerging markets. As companies struggle to adapt their logistics operations to meet the expectations of Generation Z consumers, this study provides evidence-based insights for strategic decision-making. The findings will help businesses optimize their logistics services, enhance customer experience, and build lasting relationships with the growing Generation Z consumer segment. As a result, through examining these aspects within Vietnam’s dynamic e-commerce landscape, the study not only addresses critical gaps in existing literature but also provides a foundation for future research on LSQ in emerging markets. The results hold substantial implications for academics, industry practitioners, and policymakers aiming to meet the evolving needs of digital-native consumers in competitive e-commerce environments.

## 2. Literature review

### 2.1. Theoretical background

#### 2.1.1. *Logistics services quality in e-commerce.*

Logistics service quality (LSQ) has become a cornerstone for success in the rapidly growing e-commerce sector. LSQ, as defined by Mentzer et al. [19], encompasses multiple dimensions that collectively shape the overall quality of logistics operations and directly influence customer experiences. In the e-commerce context, LSQ is integral to maintaining customer satisfaction and loyalty, given its role in ensuring seamless online shopping experiences. Do et al. [17] identified key LSQ factors such as timeliness, personal contact quality, and order accuracy, which significantly impact customer satisfaction. Akil and Ungan [16] further emphasized dimensions like order condition, discrepancy handling, and timeliness, which enhance customer perceptions of service reliability. Notably, Zia et al. [18] highlighted the convenience of return policies as a critical factor influencing customer satisfaction in the e-commerce domain. Additionally, the weight of LSQ dimensions may vary across cultural contexts, with research in Western markets emphasizing return policies, whereas studies in emerging markets underscore the role of cash-on-delivery reliability and real-time tracking [20]. These contradictions suggest that a one-size-fits-all LSQ model may be inadequate and that regional and demographic-specific adaptations are necessary.

Among the dimensions of LSQ, timeliness refers to the ability to deliver orders within the promised timeframe, a crucial expectation in the competitive online retail environment [21]. Personal contact quality, which captures the professionalism and problem-solving abilities of customer service representatives, directly affects customer satisfaction during service interactions [22]. Order accuracy, ensuring that the correct products are delivered in accordance with customer specifications, remains a fundamental expectation for e-commerce logistics [19]. Additionally, order condition pertains to the quality and integrity of products upon delivery, while order discrepancy handling involves resolving issues such as wrong deliveries or damaged goods effectively [15]. Convenience of returns, an increasingly valued dimension, reflects the ease with which customers can return products, fostering a positive post-purchase experience [18].

In Vietnam’s dynamic e-commerce sector, LSQ has gained prominence, particularly among Generation Z consumers who demonstrate higher expectations for speed, accuracy, and overall service quality compared to older generations [5]. The relationship between LSQ and customer satisfaction has been well-established in literature, with studies indicating that superior logistics service quality positively influences customer trust and satisfaction [7], which in turn enhances repurchase intentions and e-WOM [23].

#### 2.1.2. *Repurchase intention.*

Repurchase intention refers to a customer’s willingness to buy a product or service from the same retailer or seller in the future [24]. It is a critical indicator of customer loyalty and the long-term success of a business, especially in the competitive e-commerce industry [25]. According to Laia and Handini [26], repurchase intention stems from a positive evaluative judgment about the product or service quality that leads to customer satisfaction. In e-commerce, logistics service quality (LSQ) plays a vital role in shaping repurchase intentions because it directly impacts the overall shopping experience [27].

Logistics attributes, such as timeliness, order accuracy, and order discrepancy handling, significantly influence customers’ perceptions of service quality [28], which in turn fosters trust and satisfaction—two key determinants of repurchase intentions. Arabelen and Kaya [29] highlighted that service reliability and responsiveness are essential dimensions of service quality, particularly for logistics operations in online shopping contexts. Generation Z, characterized by their tech-savviness and high expectations for speed and accuracy, particularly values these logistics attributes, which influence their loyalty decisions [5]. Nevertheless, recent studies suggest that convenience alone may not be sufficient to secure long-term repurchase intentions. Owusu et al. [30] found that service failure recovery mechanisms play a crucial role in moderating the relationship between LSQ and repurchase behavior. This challenges traditional LSQ frameworks, which often focus primarily on preventive measures rather than reactive strategies.

Studies have shown that when customers trust an e-commerce platform and are satisfied with its logistics services, they are more likely to exhibit repurchase behaviors. For example, research by Le et al. [27] demonstrated a direct relationship between logistics service quality, trust, and repurchase intentions in online retail settings. Similarly, Peng et al. [31] emphasized the role of convenient return policies in enhancing customer retention, reinforcing the importance of LSQ in driving repeat purchases. Collectively, these findings highlight that investing in high-quality logistics services is a strategic priority for e-commerce businesses aiming to foster repurchase behaviors, especially among younger consumers.

#### 2.1.3. *Electronic word of mouth (eWOM).*

Electronic word of mouth (eWOM) refers to the sharing of opinions, experiences, and reviews by consumers through digital platforms, such as social media, forums, and e-commerce websites [32]. Unlike traditional word of mouth, eWOM has a broader reach and faster dissemination, making it a powerful tool for influencing consumer behavior and purchase decisions [33]. In the context of logistics service quality, eWOM can be a direct outcome of customer satisfaction and trust. High LSQ often leads to increased customer trust and satisfaction, which, in turn, motivates customers to share their positive experiences online [28]. Tandon et al. [34] observed that customers who trust and are satisfied with logistics services are more likely to engage in eWOM, thereby enhancing the retailer’s reputation. Generation Z, known for their digital engagement and reliance on peer reviews, is particularly influential in the dissemination of eWOM. When they encounter superior logistics services, this group actively shares their positive feedback on online platforms, influencing their peers’ purchasing decisions [35]. However, the relationship between LSQ and eWOM is not always straightforward. While most studies suggest a positive correlation, Abutar and Wuisan [36] and Boldureanu et al. [37] found that customers who experience minor service failures but receive outstanding recovery efforts tend to generate even more positive eWOM than those who face no issues at all. This paradoxical effect suggests that businesses should not only aim for flawless LSQ but also invest in robust recovery mechanisms to turn service failures into opportunities for enhancing brand image.

Recent studies have underscored the mediating role of customer satisfaction in fostering eWOM. For instance, Ruiz-Alba et al. [38] found that satisfied customers are more likely to share positive reviews and recommendations on digital platforms. Trust also emerges as a pivotal factor in fostering eWOM. Kim and Kim [39] highlighted trust as a key precursor, emphasizing that operational trustworthiness instills a sense of reliability, encouraging customers to share their positive experiences with their networks. In the Vietnamese e-commerce market, eWOM has a particularly pronounced impact due to the rising digital engagement of Generation Z [40]. This demographic, characterized by its active online presence, frequently uses platforms such as Facebook, TikTok, and Instagram to share their shopping experiences. These platforms serve as powerful channels through which young consumers influence the purchasing decisions of their peers [41,42]. Therefore, ensuring high logistics service quality not only promotes repeat purchases but also enhances brand advocacy by encouraging the dissemination of favorable eWOM.

### 2.2. Hypothesis development

#### 2.2.1. Timeliness.

Timeliness in logistics is a fundamental pillar for building and maintaining customer trust in e-commerce operations [43]. Recent research demonstrates that punctual delivery services play a critical role in shaping trust, particularly among Generation Z consumers who value efficiency and reliability in their purchasing experiences [5]. This demographic is notably sensitive to delivery timing, with on-time deliveries acting as a key indicator of trustworthiness when evaluating e-commerce sellers [44]. Moreover, consistent delivery performance has been shown to enhance customer confidence by signaling the seller’s operational competence and commitment to service quality [45]. Despite the strong empirical support for the role of timeliness, some studies offer divergent perspectives regarding its direct influence on trust. While timely delivery is typically associated with reliability, other service attributes—such as effective problem resolution and proactive customer support—have also been identified as significant contributors to trust development [46]. Additionally, in developing economies where logistical infrastructure remains underdeveloped, the direct influence of timeliness may be diminished. In these contexts, structural constraints and inconsistent service execution may moderate the relationship between punctual delivery and consumer trust, suggesting a need for a more context-sensitive approach to evaluating LSQ dimensions [47].

The importance of timeliness is further amplified in the e-commerce sector, where rapid urbanization and digital transformation have elevated consumer expectations regarding delivery speed [48]. Ravula [49], for instance, found that timely deliveries significantly enhance customers’ overall perception of service quality, which subsequently fosters greater trust in e-commerce platforms. When customers consistently receive their orders as promised or ahead of schedule, it cultivates a sense of reliability and competence, both of which are crucial for trust formation [43]. This is particularly significant for Generation Z consumers, whose purchase decisions are strongly influenced by prior service experiences and the perceived efficiency of delivery processes [50]. Thus, timeliness not only contributes to immediate trust but also supports the development of long-term customer relationships, positioning itself as a critical competitive advantage in the digital marketplace. Based on these literature review, the hypothesis H1 was proposed as follow:

**H1:** Timeliness has a significant positive influence on customer trust.

The influence of timeliness on customer satisfaction extends beyond functional convenience, encompassing emotional and psychological dimensions of the shopping experience [51]. Do et al. [5] indicated that prompt delivery services contribute significantly to customer satisfaction, particularly among consumers who value immediate gratification. Research by Kawa and Światowiec-Szczepańska [52] demonstrated that customers receiving their orders within the promised timeframe consistently report higher levels of satisfaction, highlighting the critical role of delivery promptness in shaping the customer experience. Nevertheless, in highly competitive e-commerce markets, customers may tolerate minor delivery delays if the overall service experience, such as real-time tracking, flexible delivery options, or superior customer support, is well managed [53]. This highlights a research gap in understanding how the interplay of different service quality dimensions influences satisfaction levels in e-commerce logistics.

In the fast-evolving e-commerce sector, delivery speed has become a key differentiator among service providers. Bhatnagr and Rajesh [54] observed that Generation Z consumers, in particular, regard timeliness as a primary factor influencing satisfaction, reflecting their elevated expectations in the digital shopping landscape. This relationship can be explained through the expectation-disconfirmation theory, which posits that when service performance meets or exceeds customer expectations, it creates a positive disconfirmation effect, enhancing satisfaction [55,56]. Furthermore, timely deliveries contribute to an overall positive shopping experience by reinforcing perceptions of reliability and efficiency, and thereby, increasing satisfaction level [57]. When consumers receive their orders on time, they are more likely to perceive the e-commerce seller as reliable and efficient, which directly correlates with higher satisfaction levels [58]. Therefore, ensuring timely order fulfillment is essential for enhancing customer satisfaction and fostering loyalty in this competitive market. Based on these literature review, the hypothesis H2 was proposed as follow:

**H2:** Timeliness has a significant positive influence on customer satisfaction.

#### 2.2.2. Personal contact quality.

Personal contact quality plays a crucial role in establishing customer trust within e-commerce logistics sector, where interpersonal interactions between service providers and customers significantly influence perceptions of reliability and commitment [59]. Study by Kumra and Sharma [60] emphasize that the professionalism and empathy displayed during customer interactions significantly impact trust levels. Similarly, Meng et al. [61] found that effective personal contact serves as a trust-building mechanism, providing reassurance and demonstrating commitment to customer service excellence. This factor is particularly relevant for Generation Z consumers, who value authentic and responsive communication in their interactions with e-commerce sellers [62]. For this demographic, the quality of personal contact acts as a critical indicator of a seller’s trustworthiness. Hiezl and Gyurácz-Németh [63] further assert that Generation Z customers evaluate service providers’ trustworthiness based on the quality of personal interactions, with positive experiences leaving a lasting impression. Likewise, Soleimani [64] confirms that when customers encounter attentive and respectful service, they are more likely to feel valued and understood, leading to heightened trust levels. However, some studies argue that personal contact quality may not always have a direct influence on trust, particularly in cases where automated services and self-service options dominate e-commerce interactions [65]. Therefore, there is a need for in-depth assessments of the impact of personal contact quality on trust in different contexts.

In the Vietnamese context, where societal norms emphasize collective relationships, the role of personal contact quality in trust formation is amplified. Trust is deeply rooted in interpersonal dynamics, making professional and empathetic communication even more essential [66]. When customers receive attentive service, they perceive the e-commerce seller as reliable and dedicated to fulfilling their commitments [67]. This perception strengthens their confidence and trust in the service provider, fostering long-term loyalty. Collectively, these findings underline the importance of personal contact quality as a foundational element in building and maintaining trust in e-commerce logistics. Based on these literature review, the hypothesis H3 was proposed as follow:

**H3:** Personal contact quality has a significant positive influence on customer trust.

The influence of personal contact quality on customer satisfaction is critical, especially in the e-commerce sector, where interaction often occurs through digital channels [14]. High-quality personal contact is characterized by clarity of communication, timely responsiveness, and personalized attention to customer needs [29]. Research has demonstrated that effective personal interactions not only enhance the customer experience but also play a pivotal role in resolving queries and addressing concerns in a timely manner, which is essential for fostering satisfaction [68]. Nonetheless, in highly digitalized shopping environments, some researchers suggest that the impact of personal contact quality on satisfaction may be mitigated by other factors, such as self-service convenience, chatbots, or automated support [69].

For Generation Z consumers, who favor seamless and interactive shopping experiences, the quality of personal contact can significantly impact their overall satisfaction with e-commerce services [70]. Personalization, such as tailored recommendations or proactive support, further elevates their satisfaction levels by making them feel understood and appreciated [71]. Rita et al. [72] highlight that when customers perceive personalized and empathetic communication, they are more likely to report higher levels of satisfaction. These interactions contribute to creating a positive perception of the service seller, reinforcing loyalty and encouraging repeat purchases. Based on these literature review, the hypothesis H4 was proposed as follow:

**H4:** Personal contact quality has a significant positive influence on customer satisfaction.

#### 2.2.3. Order accuracy.

Order accuracy, defined as the ability of e-commerce sellers to deliver the correct product in the right quantity as promised, is a cornerstone of customer trust in the e-commerce logistics context [16]. Unlike traditional shopping environments where customers can verify their purchases before leaving the store, e-commerce heavily relies on trust in the platform to ensure accurate order fulfillment [57]. This aspect is especially significant for Generation Z consumers, who have grown up with online shopping as a norm and possess higher expectations for service quality [54].

Accurate order fulfillment not only demonstrates operational competence but also reinforces the reliability of the e-commerce seller. Camilleri [73] emphasizes that this reliability is integral to building trust, as customers equate accuracy with the seller’s professionalism and commitment to quality. Soleimani [64] further explains that inaccuracies in order delivery can cause frustration, erode trust, and create apprehensions about future transactions. Accurate order fulfillment not only reassures customers of the seller’s credibility but also alleviates concerns regarding potential risks associated with online shopping, such as receiving incorrect or damaged items [74]. However, while Western consumers may perceive minor inaccuracies as acceptable due to flexible return policies [75], Asian consumers may view them as a sign of incompetence, leading to stronger negative reactions [76]. This highlights the need for a nuanced understanding of order accuracy’s role in shaping trust across different markets.

The importance of order accuracy is amplified for Generation Z consumers, who are known for their discerning attitudes and elevated expectations. This demographic is highly influenced by their initial experiences with a brand, with accurate order fulfillment playing a key role in building trust and securing long-term loyalty [77]. When order accuracy aligns with customer expectations, it not only assures them of the seller’s credibility but also positions the platform as a reliable choice for future transactions. Therefore, based on these literature review, the hypothesis H5 was proposed as follow:

**H5:** Order accuracy has a significant positive influence on customer trust.

The influence of order accuracy extends beyond trust and significantly impacts customer satisfaction, making it a key determinant of positive customer experiences in e-commerce [78]. Precise order fulfillment contributes to higher satisfaction levels, particularly for Generation Z consumers who demand seamless and reliable shopping experiences [79]. Research shows that discrepancies in order fulfillment, such as receiving incorrect items or incomplete shipments, lead to dissatisfaction and negative perceptions of the seller [74].

On the other hand, when customers consistently receive what they ordered, it fosters a sense of being valued and understood, which directly enhances satisfaction [80,81]. This alignment of service delivery with customer expectations creates a positive feedback loop, reinforcing satisfaction and encouraging repeat purchases [82]. For Generation Z consumers, order accuracy is not merely a convenience but an expectation that defines their overall satisfaction with the shopping experience [83]. Thus, ensuring high order accuracy is vital for shaping customer perceptions and achieving high satisfaction rates in the competitive e-commerce market. Based on these literature review, the hypothesis H6 was proposed as follow:

**H6:** Order accuracy has a significant positive influence on customer satisfaction.

#### 2.2.4. Order condition.

The condition of the product upon delivery plays a significant role in establishing customer trust in the context of e-commerce logistics [84]. Order condition refers to the state of the product when it reaches the customer, including its physical integrity and packaging quality. Research highlights that well-maintained product condition demonstrates the e-commerce seller’s reliability and commitment to quality, positively influencing customer trust [85]. Trust, defined as the consumer’s belief in a brand’s reliability and integrity, plays a key role in shaping customer perceptions and purchase decisions [86]. Secure packaging and undamaged delivery instill confidence in customers, reassuring them that the seller values their satisfaction and adheres to high operational standards [87]. This assurance fosters a positive perception of the brand and encourages repeat purchases. Singh [87] further emphasizes that consistently receiving well-packaged, undamaged goods enhances customer trust and loyalty. Similarly, Al-Muani et al. [88] explain that delivering products in excellent condition reflects the seller’s dedication to customer satisfaction, which is critical for trust-building. On the other hand, some studies argue that excessive packaging may be perceived negatively due to environmental concerns, particularly among Generation Z consumers who prioritize sustainability [89]. This suggests that while order condition is crucial, businesses must balance product protection with eco-friendly practices to maintain trust. Conversely, poor product condition—such as damage or inadequate packaging—can lead to negative customer experiences, eroding trust and discouraging future transactions [16].

For Generation Z consumers, who prioritize visual cues and quality, there’s a strong correlation between the service reliability perceived through product condition and their trust in the vendor [90]. Thus, maintaining high standards in packaging and ensuring product integrity are essential for building and sustaining trust, especially among the discerning Generation Z shoppers. Through focusing on product condition, e-commerce businesses can enhance customer satisfaction and foster long-term loyalty, crucial for success in the competitive online market. Based on these literature review, the hypothesis H7 was proposed as follow:

**H7:** Order condition has a significant positive influence on customer trust.

Beyond trust, order condition significantly impacts customer satisfaction, which is defined as the extent to which a product or service meets or exceeds customer expectations [91]. Research consistently shows that when products arrive in excellent condition, customer satisfaction increases, as the delivery meets their expectations [92,93]. This positive experience is particularly important for Generation Z, who value quality and detailed attention in the services they receive [83]. The study by Al-Adwan and Yaseen [90] also demonstrated that Generation Z consumers reported heightened satisfaction with companies that prioritize secure, protective packaging.

In addition, well-maintained order condition minimizes dissatisfaction related to product damage, reinforcing a positive experience and solidifying customer satisfaction with the e-commerce service [94]. Moreover, Tedja et al. [95] assert that receiving undamaged and well-presented products upon delivery enhances customers’ perceived value of the service, as it reflects the seller’s commitment to quality. This is especially critical for Generation Z, who are quick to share their satisfaction—or dissatisfaction—online, thereby influencing broader consumer perceptions [96]. As a result, maintaining optimal order conditions not only meets customer expectations but also fosters repeat purchases and positive feedback, further boosting overall customer satisfaction. Based on these literature review, the hypothesis H8 was proposed as follow:

**H8:** Order condition has a significant positive influence on customer satisfaction.

#### 2.2.5. Order discrepancy handling.

Order discrepancy handling is a critical factor in establishing and maintaining customer trust [97]. When a seller effectively manages and resolves issues related to incorrect, missing, or damaged items, it signals a commitment to accountability and customer care, which reassures customers about the seller’s reliability [88]. Effective discrepancy handling practices showcase the seller’s responsiveness, enhancing customers’ perception of trustworthiness and reliability [98]. Liu et al. [99] highlight that a seller’s ability to address order issues with transparency and empathy strengthens customers’ confidence, particularly among Generation Z consumers, who interpret proactive communication and rapid resolution as hallmarks of a trustworthy brand.

In the Vietnamese e-commerce market, where service reliability is a key driver of consumer loyalty, effective order discrepancy handling has been shown to positively impact trust by making customers feel valued and prioritized [88]. By addressing issues promptly and satisfactorily, companies not only build initial trust but also foster long-term loyalty and commitment. However, research from global markets reveals variations in the impact of such practices. For example, in Western markets, return-friendly policies and automated resolution systems play a crucial role in minimizing order discrepancies’ negative effects on trust [100]. In contrast, in emerging economies like Vietnam, direct customer interaction and personalized resolution approaches may play a more significant role [101]. Based on these literature review, the hypothesis H9 was proposed as follow:

**H9:** Order discrepancy handling has a significant positive influence on customer trust.

In addition to building trust, effective order discrepancy handling plays a vital role in enhancing customer satisfaction, as it helps alleviate the stress and frustration customers may feel when issues arise with their orders [16]. The ability to address these discrepancies quickly and satisfactorily directly correlates with higher satisfaction levels, as customers feel that their concerns are being addressed and respected [102]. According to Akıl and Ungan [16], positive and efficient handling of order discrepancies leads to an improved perception of the brand or service, reinforcing customers satisfaction that their needs are valued and prioritized. This is particularly significant for Generation Z customers, who, according to [103], expect fast and effective problem resolution as a standard part of their shopping experience. For this demographic, slow or inadequate handling of discrepancies can lead to dissatisfaction and a damaged brand image, while rapid and thoughtful resolution fosters positive experiences and enhances overall satisfaction [104]. Interestingly, some global studies suggest that expectations for order discrepancy resolution vary by region. Research by Asawawibul et al. [69] indicates that in developed e-commerce markets, automated chatbots and AI-driven resolution systems significantly enhance satisfaction. In contrast, consumers in developing economies may still prefer human interaction and direct customer service engagement in handling order discrepancies [105]. Thus, the effective handling of order discrepancies not only meets customer expectations but also enhances their overall satisfaction, creating a foundation for stronger relationships and greater loyalty. Based on these literature review, the hypothesis H10 was proposed as follow:

**H10:** Order discrepancy handling has a significant positive influence on customer satisfaction.

#### 2.2.6. Convenience of return.

A streamlined and accessible return process is a critical factor in building customer trust, as it reflects a seller’s dedication to resolving post-purchase concerns and ensuring customer satisfaction [106]. When a business offers flexible, clear, and hassle-free return policies, customers feel more confident in their purchasing decisions, as they know they have the option to return items if necessary [107]. This flexibility not only reduces the psychological risk associated with online shopping but also reflects a customer-centric approach that prioritizes the buyer’s needs and concerns [108].

For Generation Z consumers, who often face uncertainties in the e-commerce space, convenient return policies are especially important. Serravalle et al. [109] indicate that this demographic considers the ease of return policies a significant factor in their trust in a brand. A simple and transparent return process is perceived as an indicator of reliability, showcasing the seller’s commitment to customer care and reinforcing trust [110]. Furthermore, research by Rokonuzzaman et al., [111] emphasizes that an efficient return process helps mitigate the inherent risks of online shopping, where customers cannot physically inspect products before purchase. While some studies suggest that hassle-free returns are a trust-enhancing factor across all customer segments [112], others caution that excessively lenient return policies may signal low product quality or a lack of seller credibility [113]. These contrasting findings suggest a need for further investigation into achieving an optimal balance between return policy flexibility and perceived product reliability. Ultimately, by facilitating a seamless return experience, businesses can enhance consumers’ sense of security and strengthen perceptions of brand dependability [114]. Based on these literature review, the hypothesis H11 was proposed as follow:

**H11:** Convenience of return has a significant positive influence on customer trust.

In addition to trust, the convenience of the return process significantly impacts overall customer satisfaction by providing an effective solution to post-purchase issues [115]. When companies implement flexible and accessible return policies, they cater to customers’ expectations for efficient post-purchase support, which directly enhances satisfaction levels [116]. Such policies alleviate frustrations customers may encounter if a product fails to meet their expectations, ensuring that they have an easy means of resolving their dissatisfaction without additional inconvenience [106].

Das and Kunja [117] specifically highlight the importance of hassle-free returns for Generation Z consumers, who prioritize a seamless post-purchase experience. According to Rintamäki et al. [118], the presence of an uncomplicated return policy makes customers feel valued and understood, further enhancing their satisfaction with the brand. Similarly, Wang et al. [119] note that a straightforward return process reduces the perceived risk of online shopping, where consumers who seek reassurance in case of post-purchase issues observe that a straightforward return process reduces perceived risk in online shopping, reassuring consumers that they retain control over the transaction even if issues arise. Such convenience reflects the brand’s responsiveness to customer needs, leading to increased satisfaction and loyalty. Based on these literature review, the hypothesis H12 was proposed as follow:

**H12:** Convenience of return has a significant positive influence on customer satisfaction.

#### 2.2.7. Customer trust.

Customer trust is a fundamental determinant of customer satisfaction, especially in e-commerce [120]. Trust in the logistics aspect of the service assures customers that their purchases will be delivered reliably and accurately, which enhances their overall satisfaction with the e-commerce experience [121]. When customers have confidence in a seller’s logistics capabilities, they are less worried about potential delivery issues, enabling them to focus more on the quality and value of the purchased products rather than anxieties related to order fulfillment [98]. This reduction in perceived risk is particularly impactful in the Vietnamese e-commerce market, where consumer trust in the logistics process can strongly influence satisfaction with the seller [122]. For Generation Z consumers, seamless and transparent logistics are especially valued, with timely delivery, accurate tracking, and effective communication about order status enhancing their trust and satisfaction [123–125]. By meeting customer expectations through reliable logistics, sellers can foster a positive perception of their brand, reinforcing customer satisfaction and long-term loyalty [126]. Based on these literature review, the hypothesis H13 was proposed as follow:

**H13:** Customer trust has a significant positive influence on customer satisfaction.

In the e-commerce landscape, trust is a powerful driver of repurchase intention, as customers are more likely to return to sellers they perceive as reliable, particularly in terms of logistics services [25]. Trust in the seller’s logistics quality—such as prompt deliveries, order accuracy, and efficient resolution of discrepancies—build a sense of security, making customers more inclined to choose the same seller for future purchases [127]. Le et al [27] and Mallieswari et al [35] emphasize that Generation Z consumers, who are highly value-driven and sensitive to seamless service, consider trust in logistics a major factor in their decision to repurchase from a particular seller. A consistent and reliable logistics experience not only assures customers of the seller’s capability but also strengthens their loyalty, reducing the likelihood of switching to competitors [128]. Moreover, in the competitive e-commerce environment, where customers can easily switch between sellers, the trust cultivated through dependable logistics services can be a distinguishing factor [129]. By establishing trust, sellers not only encourage repeat purchases but also reduce customer attrition, creating a loyal customer base that is more resilient to alternative offerings [130,131]. Based on these literature review, the hypothesis H14 was proposed as follow:

**H14:** Customer trust has a significant positive influence on repurchase intention.

Trust plays a crucial role not only in boosting customer satisfaction and repurchase intentions but also in encouraging customers to share their positive experiences through electronic word of mouth (eWOM) [132]. When customers trust a seller’s logistics capabilities, such as timely delivery, accurate tracking, and swift issue resolution, they are more inclined to advocate for the brand online, sharing their experiences whether through social media, review platforms, or personal networks [133,134]. This effect is particularly relevant for Generation Z, a demographic that frequently uses eWOM to express satisfaction and loyalty [135,136]. Research indicates that a strong level of trust not only leads customers to feel secure in their purchases but also motivates them to promote the brand voluntarily, contributing to its positive reputation in a competitive e-commerce landscape [128,137]. Furthermore, Quaye et al [138] highlight that trust encourages brand advocacy, as customers feel confident in recommending sellers who consistently meet or exceed their expectations. Therefore, building trust is an effective strategy for generating positive, organic eWOM, which boosts brand visibility and attracts new customers. Based on these literature review, the hypothesis H15 was proposed as follow:

**H15:** Customer trust has a significant positive influence on electronic word of mouth.

#### 2.2.8. Customer satisfaction.

Customer satisfaction is a pivotal factor in driving repurchase intentions, as it strengthens customer loyalty and fosters long-term relationships with service providers [139]. When customers experience satisfaction, they develop positive associations with the brand, which enhances their preference and reduces their likelihood of switching to competitors [140,141]. The relationship between satisfaction and repurchase intention is notably pronounced among Generation Z consumers, who prioritize consistent service quality and positive experiences in their buying decisions [142]. In e-commerce, satisfaction is significantly important for Generation Z, who rely heavily on digital platforms and lack the opportunity to physically evaluate products. For these consumers, trust and satisfaction in service quality are critical determinants of repurchase behavior [143,144]. Moreover, Kusumawardani and Hastayanti [59] and Lin et al [14] highlight that customer satisfaction with logistics service quality—especially in aspects such as delivery speed and order accuracy—directly influences the likelihood of repeat purchases. A reliable and satisfying shopping experience creates a positive feedback loop, as customers are more likely to return to sellers they perceive as dependable and aligned with their expectations [59]. Based on these literature review, the hypothesis H16 was proposed as follow:

**H16:** Customer satisfaction has a significant positive influence on repurchase intention.

In addition to fostering repurchase intentions, customer satisfaction also plays a key role in encouraging electronic word of mouth (eWOM) [132]. Satisfied customers are more likely to share their positive experiences, becoming informal brand advocates who influence potential customers [145]. Generation Z consumers, known for their high engagement on digital and social media platforms, are particularly likely to express satisfaction through eWOM by leaving reviews, providing product ratings, and recommending services to their social circles [146,147]. This demographic values authenticity and peer recommendations, often viewing customer-generated content as more credible than conventional marketing messages [148]. In e-commerce, where trust in peer recommendations remains vital, satisfied customers amplify brand credibility and visibility by generating organic, positive content that attracts new buyers [144]. The tendency of satisfied customers to advocate for brands they trust is a powerful asset, particularly in competitive markets where word of mouth serves as a critical tool for differentiation [147]. Through their online engagement, satisfied customers enhance brand reputation and reach, solidifying the brand’s position in the market. Based on these literature review, the hypothesis H17 was proposed as follow:

**H17:** Customer satisfaction has a significant positive influence on electronic word of mouth.

#### 2.2.9. Repurchase intention.

Repurchase intention reflects a customer’s willingness to continue engaging with a brand, which not only drives repeat purchases but also encourages customers to share their positive experiences through electronic word of mouth (eWOM) [149]. When customers are satisfied and committed to a brand, their intention to repurchase often leads them to advocate for the brand, reinforcing its reputation [150]. This tendency is particularly evident among Generation Z consumers, who actively communicate their brand loyalty and share product insights on social media platforms, influencing the perceptions of their peers [151,152].

Research highlights the persuasive power of eWOM generated by returning customers. Rachbini et al [153] found that customers with strong repurchase intentions often provide high-value eWOM, sharing testimonials that reflect their trust, satisfaction, and commitment to the brand. These contributions signal a higher level of trust and satisfaction, which enhances the persuasive impact of the eWOM they generate. Loyal customers who engage in eWOM effectively validate their purchasing decisions and serve as ambassadors for the brand, helping to attract new customers while reinforcing the loyalty of existing ones [154]. This cyclical relationship between repurchase intention and eWOM ensures that brands not only retain their customers but also expand their reach through organic, customer-driven promotion. Based on these literature review, the hypothesis H18 was proposed as follow:

**H18:** Repurchase intention has a significant positive influence on electronic word of mouth.

### 2.3. Research framework

**Fig 1:**
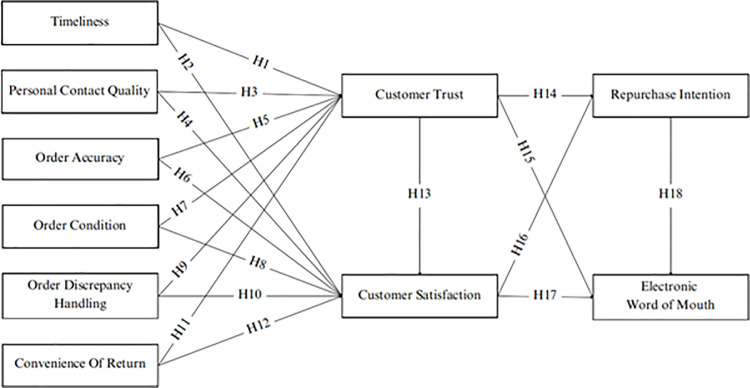
The proposed research framework.

The theoretical framework for this study (Figure 1) explores the interconnections between logistics service quality (LSQ), customer trust, customer satisfaction, repurchase intention, and electronic word of mouth (eWOM) within the e-commerce landscape. This framework underscores the importance of LSQ in shaping customer behaviors, as it directly influences both trust and satisfaction. Specifically, LSQ is conceptualized through six key dimensions: timeliness, personal contact quality, order accuracy, order condition, order discrepancy handling, and the convenience of returns. Trust plays a pivotal role in the framework, serving as both a direct and indirect driver of behavioral outcomes. It directly influences customer satisfaction and also shapes repurchase intentions and eWOM, demonstrating its central role in fostering long-term relationships and advocacy. Similarly, satisfaction acts as a mediating variable, translating the effects of LSQ and trust into customer loyalty and positive eWOM behavior.

The model underscores the asymmetrical and interconnected nature of these relationships rather than viewing them as strictly linear or direct. LSQ directly enhances trust and satisfaction, which, in turn, mediates its impact on repurchase intention and eWOM. This layered approach highlights the significance of LSQ in shaping customer perceptions and behaviors, showcasing its vital role in driving trust, satisfaction, and advocacy in the highly competitive and dynamic e-commerce context.

## 3. Methodology

### 3.1. Participants

This study investigates the influence of logistics service quality (LSQ) on repurchase intention and electronic word-of-mouth (eWOM) behavior within the e-commerce industry, focusing on Generation Z individuals in Vietnam. Defined as those born between 1995 and 2010, Generation Z represents digital natives who have grown up in the internet age and are characterized by their active engagement with social media and e-commerce platforms [155]. This demographic’s familiarity with digital technologies and online shopping behaviors makes them an ideal target group for examining LSQ’s impact on consumer attitudes and behaviors.

The study’s sample consisted of 551 initial survey responses, collected from individuals with prior online shopping experience to ensure relevance and alignment with the research objectives [156]. After rigorous data screening to exclude responses with no shopping experience on e-commerce platforms, as well as incomplete, illogical, or hastily completed responses, 495 valid responses were retained for analysis [157]. The demographic information of survey participants, as detailed in Table 1, revealed that the majority of respondents (78.2%) were aged between 18 and 22, reflecting the younger segment of Generation Z, followed by a smaller proportion aged 23–26 (13.1%) and 27–29 (8.7%). Gender distribution showed that 55.4% of participants were female, 42.8% were male, and 1.8% preferred not to specify. Regarding online shopping behavior, most participants reported a monthly income of less than 5 million VND (59.2%) or from 5 to 10 million VND (20%). The majority also indicated that they shopped online one to two times per month, typically spending less than 1 million VND per month. Shopee and TikTok were identified as the most frequently used e-commerce platforms.

**Table 1:** Respondents’ profile.

Demographic Information		Frequency
Gender	Male	212
	Female	274
	Not to specify	9
Age	18 to 22 years old	387
	23 to 26 years old	65
	27 to 29 years old	43
Monthly income	Less than 5 million VND	293
	From 5 to 10 million VND	99
	From 10 to 20 million VND	52
	From 20 to 30 million VND	39
	More than 30 million	12
Monthly spending on e-commerce platforms	Less than 1 million VND	202
	From 1 to 3 million VND	176
	From 3 to 5 million VND	70
	From 5 to 7 million VND	26
	More than 7 million VND	20
Monthly shopping frequency on e-commerce platforms	Less than once a month	55
	1 - 2 times per month	214
	3 - 5 times per month	138
	More than 5 times per month	88
Preferred online shopping platform (Multiple options)	TikTok	370
	Shopee	452
	Lazada	77
	Tiki	45

The demographic and behavioral characteristics of the sample confirm its suitability for examining the impact of LSQ on customer attitudes and behaviors in e-commerce. The strong representation of Generation Z consumers with substantial online shopping experience ensures that the findings will be both relevant and meaningful in understanding this demographic’s unique engagement with e-commerce platforms.

### 3.2 . Instruments

A structured online-based questionnaire was employed as the main tool for collecting data in this study. Its design was guided by theoretical principles and supported by findings from empirical research. The questionnaire was organized into three sections to ensure comprehensiveness and alignment with the study’s objectives. The first section functioned as a screening tool to verify participant eligibility, reducing the risk of including unsuitable respondents in the online survey [59]. The second section focused on gathering respondents’ demographic and personal information, such as age, gender, income, and online shopping behaviors, providing a detailed profile of the respondents. The final section evaluated the research model using a multi-item approach, which minimizes individual measurement errors and enhances the reliability and validity of constructs [158].

The final section consisted of 37 closed-ended items designed to measure 10 latent constructs using a five-point Likert scale ranging from “1 = strongly disagree” to “5 = strongly agree,” as detailed in Appendix. Logistics Service Quality (LSQ), the central independent variable, was conceptualized through six dimensions. Specifically, Order Condition (OC) and Order Accuracy (OA) were each assessed using three items sourced from Akıl and Ungan [16] and Bienstock and Royne [159]. Convenience of Return (CR) was measured using four items derived from Kawa and Światowiec-Szczepańska [52]. Timeliness (TL), Personal Contact Quality (PCQ), and Order Discrepancy Handling (ODH) were each evaluated using three items adapted from Akıl and Ungan [16], Bienstock et al. [160], Jiang et al. [161], Mentzer et al. [19] and Lin et al. [14]. Two mediating variables, Customer Trust (CT) and Customer Satisfaction (CS), were included to examine indirect effects. CT was measured using four items adapted from Falahat et al. [162], while CS was assessed with four items based on Kim et al. [163], Yang et al. [164], and Correa et al. [165]. The two outcome variables, Repurchase Intention (RI) and Electronic Word-of-Mouth (eWOM), were measured using five items adapted from Hsu et al. [166] and Wijaya et al. [167] and four items from Mim et al. [168], respectively. All items were carefully adapted and contextualized from validated sources to ensure their alignment with the study’s theoretical framework and relevance to the e-commerce context.

A pilot survey was conducted to assess the instrument’s feasibility and identify potential design issues before the main data collection phase [169]. A sample of 30 respondents participated in the pilot study, which is considered sufficient for detecting methodological concerns [170]. The results showed that all constructs achieved Cronbach’s alpha values above 0.7, indicating satisfactory internal consistency [171]. Feedback from participants confirmed that the survey items were clear, relevant, and suitable for the target population, validating the appropriateness of the questionnaire for the main study. This rigorous pilot testing ensured the reliability and validity of the research instrument, laying a strong foundation for subsequent data collection and analysis.

### 3.3. Data collection

This study adopted a quantitative research approach, using a structured, questionnaire-based survey to collect primary data [172]. The survey was designed to enable respondents to independently express their opinions on structured questions and statements without external influence, ensuring data accuracy and integrity [173]. Participation in the survey was entirely voluntary, with informed consent obtained before granting access to the questionnaire. This practice adhered to ethical research standards and underscored participant rights and confidentiality [174].

The survey was administered via Google Forms, selected for its user-friendly interface and compatibility across diverse devices, which made it convenient for respondents to participate [175]. To maximize participation and ensure a diverse respondent base, the survey link was disseminated through popular social media platforms. A non-probability convenience sampling method was employed, chosen for its efficiency in accessing a broad pool of respondents within the target demographic quickly and cost-effectively [176,177]. This method was particularly suitable for engaging Generation Z, who are active on digital platforms and align with the study’s focus.

The sample size for this study was determined based on Hair et al.’s [178] recommendation, which suggests a minimum 1:10 ratio of indicators to sample size, leading to a target of at least 370 valid responses. To ensure adequate representation and reduce sampling error, as highlighted by Bryman’s [179], a larger sample size was pursued. The research began with a pilot study conducted from September 4 to September 5, 2024, yielding 30 responses to test the survey instrument. Following this, the empirical study was carried out between September 9 and September 30, 2024. During this period, the online survey was distributed through social media platforms, including Facebook, Zalo, and TikTok. A total of 551 responses were received. After thoroughly reviewing the submissions and excluding incomplete or ineligible responses, 495 valid responses were retained, resulting in a high valid response rate of 89.8%.

Ethical considerations were meticulously observed throughout the research process. At the beginning of the survey, respondents were required to provide explicit written consent by ticking a checkbox, confirming their voluntary participation and their agreement to the use of their responses for research purposes. The survey included a detailed explanation of the study’s objectives and emphasized the anonymity and confidentiality of participant data. Ethical approval for the study was granted by the Board of Directors at FPT Can Tho University, Vietnam (Approval No. 20240603.08). All procedures strictly adhered to ethical guidelines for research involving human subjects, ensuring participant privacy and protection.

### 3.4. Data analysis

Data from the survey were analyzed using Partial Least Squares Structural Equation Modeling (PLS-SEM), a component-based SEM method that evaluates both the measurement and structural models [180]. PLS-SEM was selected for its robustness in handling complex models, its suitability for exploratory research, and its capacity to yield reliable results with small to medium sample sizes [181].

The data analysis followed a rigorous two-stage process as recommended by Anderson and Gerbing [182]. The first stage focused on the measurement model to ensure reliability and validity of the constructs. Reliability was assessed using Cronbach’s Alpha (CA) and Composite Reliability (CR), while convergent validity was confirmed by calculating the Average Variance Extracted (AVE). Discriminant validity was tested through the Fornell-Larcker criterion and the Heterotrait-Monotrait Ratio (HTMT) to confirm that each construct was unique and appropriately correlated with others. In the second stage, the structural model was analyzed to evaluate the hypothesized relationships between latent constructs. Bootstrapping techniques were applied to assess the significance of path coefficients. Prior to this, Variance Inflation Factors (VIF) were calculated to detect and address any potential multicollinearity among predictor variables. Model explanatory power was assessed using R² values, and predictive relevance was evaluated through Q² statistics, while the effect size (f²) was examined to understand the relative impact of predictor variables, providing a comprehensive understanding of the model’s performance [181,183].

This systematic analytical approach ensured the robustness and credibility of the findings, supporting the study’s contribution to the theoretical and practical understanding of logistics service quality’s impact on consumer behavior in e-commerce.

## 4. Results

### 4.1. Measurement model assessment

#### 4.1.1. *Measurement model robustness.*

The robustness of the measurement model was evaluated to ensure internal consistency, convergent validity, and discriminant validity of the constructs, following established guidelines for model assessment [180]. Reliability was examined through indicator reliability and internal consistency. As shown in Table 2, the outer loadings of indicators ranged from 0.718 to 0.838, surpassing the acceptable threshold of 0.6 [184], confirming their statistical significance and reliability. Internal consistency was further evaluated using Cronbach’s Alpha, indicates the degree to which items within a construct are interrelated, ensuring they function as a unified and cohesive entity [185], with values between 0.711 and 0.808, demonstrating sufficient reliability. Composite Reliability (CR), which reflects the extent to which indicators represent their respective latent constructs [186], ranged from 0.825 to 0.872, all exceeding the recommended threshold of 0.7 [180,187]. These results confirm that the model is reliable and exhibits strong internal consistency.

**Table 2:** Construct reliability and validity.

Constructs	Items	Loadings	Cronbach’s Alpha	Composite Reliability	VIF
**Timeliness (TL)**	TL1: “The time between placing an online order and receiving the delivery is short.”	0.738	0.718	0.825	1.377
	TL2: “The goods are delivered on the promised date.”	0.738			1.330
	TL3: “If there’s a delay, the logistics provider quickly reschedules the delivery.”	0.737			1.309
	TL4: “The rate of non-compliance with set delivery times is very low.”	0.731			1.367
**Personal Contact Quality (PCQ)**	PCQ1: “The logistics service employees possess adequate knowledge and experience to handle inquiries and problems competently.”	0.812	0.711	0.839	1.450
	PCQ2: “The logistics service employees consistently display a positive attitude when addressing my concerns.”	0.817			1.425
	PCQ3: “The logistics service employees maintain a courteous demeanor when dealing.”	0.759			1.324
**Order Accuracy (OA)**	OA1: “Deliveries consistently contain the correct items.”	0.791	0.741	0.853	1.415
	OA2: “The quantity of items delivered is always accurate.”	0.828			1.520
	OA3: “Delivered products match the order specifications (e.g., model, color).”	0.816			1.497
**Order Condition (OC)**	OC1: “The product I ordered was delivered with appropriate protection.”	0.782	0.717	0.840	1.268
	OC2: “Product is rarely damaged due to shipping method.”	0.795			1.566
	OC3: “Product is rarely damaged due to handling by the shipping unit.”	0.818			1.533
**Order Discrepancy Handling (ODH)**	ODH1: “It is easy to report order discrepancies to the seller.”	0.784	0.725	0.845	1.379
	ODH2: “The seller offers satisfactory solutions for order discrepancies.”	0.787			1.420
	ODH3: “Overall, the seller provides strong support in resolving product issues.”	0.838			1.506
**Convenience of Return (CR)**	CR1: “I can return products for free when I shop online.”	0.766	0.746	0.840	1.519
	CR2: “The return process is easy when I shop online.”	0.771			1.442
	CR3: “I can return products within a specified time when I shop online.”	0.743			1.417
	CR4: “I am able to return used products in some cases when I shop online.”	0.733			1.363
**Customer Trust (CT)**	CT1: “I believe the e-commerce logistics service is trustworthy.”	0.746	0.765	0.850	1.420
	CT2: “I believe the e-commerce logistics service keeps promises and commitments.”	0.772			1.517
	CT3: “I believe the e-commerce logistics service always has my best interests in mind.”	0.750			1.417
	CT4: “I believe the e-commerce logistics service meets my expectations.”	0.795			1.559
**Customer Satisfaction (CS)**	CS1: “I am satisfied with the e-commerce retailer’s logistics services.”	0.718	0.719	0.826	1.320
	CS2: “The logistics services for e-commerce purchases fulfill my demand.”	0.743			1.374
	CS3: “The logistics experience I had with online retailers was exactly what I needed.”	0.753			1.388
	CS4: “Using the logistics services offered by retailers on e-commerce platforms was the right one.”	0.732			1.343
**Repurchase Intention (RI)**	RI1: “I am highly likely to continue purchasing products from this e-retailer in the future.”	0.742	0.808	0.867	1.532
	RI2: “I intend to continue shopping with this e-retailer rather than switch to others.”	0.782			1.687
	RI3: “I will be back to repurchase products from this e-retailer in the future.”	0.718			1.440
	RI4: “I prefer to buy products from this e-retailer rather than other online stores.”	0.754			1.535
	RI5: “I will prioritize this e-retailer for future purchases.”	0.764			1.623
**Electronic Word-of-Mouth (EW)**	EW1: “I will recommend this e-retailer to others through e-commerce platforms.”	0.794	0.804	0.872	1.617
	EW2: “I will speak of the good sides of this e-retailer on social media.”	0.766			1.558
	EW3: “I will be proud to say to others that I am a customer of this e-retailer.”	0.787			1.621
	EW4: “I will speak favorably of this e-retailer to others.”	0.829			1.830

*Note.* VIF = Variance Inflation Factor

#### 4.1.2. *Convergent and Discriminant Validity.*

To evaluate convergent validity, which indicates the extent to which items within the same construct are closely related [162], the Average Variance Extracted (AVE) was utilized. AVE represents the proportion of variance in the indicators explained by the latent construct. In this research model, all constructs had AVE values exceeding the threshold of 0.5, as shown in Table 3, indicating that more than half of the variance in the indicators was captured by their respective constructs. This demonstrates strong convergent validity [155].

**Table 3:** Convergent validity.

Constructs	Average Variance Extracted (AVE)
TL	0.541
PCQ	0.634
OA	0.659
OC	0.637
ODH	0.645
CR	0.568
CT	0.587
CS	0.543
RI	0.566
EW	0.631

*Notes* TL = Timeliness; PCQ = Personal contact quality; OA = Order accuracy; OC = Order condition; ODH = Order discrepancy handling; CR = Convenience of return; CT = Customer trust; CS = Customer satisfaction; RI = Repurchase intention; EW = Electronic word-of-mouth.

Discriminant validity examines whether items effectively differentiate between constructs and measure distinct concepts [188]. Discriminant validity was assessed using two methods: the Fornell and Larcker criterion and the Heterotrait-Monotrait Ratio (HTMT). According to the Fornell and Larcker criterion, which compares the square root of the AVE for each latent variable to its correlations with other variables. Discriminant validity is established when the square root of the AVE for each construct exceeds its correlations with other constructs [189]. Table 4 shows that the square root of the AVE for all constructs surpassed their respective correlations, providing strong evidence of discriminant validity.

**Table 4:** Discriminant validity (Fornell-Larcker Criterion).

	CR	CS	CT	EW	OA	OC	ODH	PCQ	RI	TL
**CR**	0.753									
**CS**	0.564	0.737								
**CT**	0.632	0.650	0.766							
**EW**	0.548	0.547	0.633	0.794						
**OA**	0.495	0.449	0.547	0.493	0.812					
**OC**	0.507	0.387	0.528	0.543	0.582	0.798				
**ODH**	0.656	0.451	0.579	0.531	0.456	0.460	0.803			
**PCQ**	0.609	0.541	0.574	0.544	0.585	0.498	0.565	0.796		
**RI**	0.607	0.536	0.620	0.667	0.477	0.488	0.578	0.489	0.752	
**TL**	0.534	0.480	0.505	0.490	0.556	0.485	0.484	0.611	0.446	0.736

*Notes* TL = Timeliness; PCQ = Personal contact quality; OA = Order accuracy; OC = Order condition; ODH = Order discrepancy handling; CR = Convenience of return; CT = Customer trust; CS = Customer satisfaction; RI = Repurchase intention; EW = Electronic word-of-mouth.

The HTMT criterion, which evaluates discriminant validity using a multi-trait, multi-method approach, further corroborated these findings [190]. Based on the recommendation by Henseler et al. [190], issues with discriminant validity arise when HTMT values are excessively high. For structural models where constructs are conceptually similar, a threshold of 0.9 is considered acceptable. However, in models where constructs are more distinct, a stricter criterion of less than 0.85 is advised to ensure adequate discriminant validity. As shown in Table 5, the HTMT values for most construct pairs range from 0.533 to 0.834, meeting the stricter threshold of <0.85. For conceptually similar constructs, such as Customer Trust (CT) and Customer Satisfaction (CS), Personal Contact Quality (PCQ) and Timeliness (TL), or Convenience of Return (CR) and Order Discrepancy Handling (ODH), the values range from 0.853 to 0.890, which remain below the 0.9 threshold deemed acceptable. This confirms that discriminant validity is established for this study.

**Table 5:** Discriminant validity (Heterotrait-Monotrait Ratio).

	CR	CS	CT	EW	OA	OC	ODH	PCQ	RI	TL
**CR**										
**CS**	0.771									
**CT**	0.833	0.876								
**EW**	0.708	0.717	0.805							
**OA**	0.665	0.614	0.729	0.639						
**OC**	0.688	0.533	0.703	0.708	0.796					
**ODH**	0.890	0.622	0.778	0.692	0.620	0.630				
**PCQ**	0.834	0.755	0.777	0.718	0.806	0.694	0.784			
**RI**	0.783	0.703	0.788	0.826	0.616	0.639	0.751	0.642		
**TL**	0.727	0.667	0.677	0.645	0.763	0.668	0.669	0.853	0.585	

*Notes* TL = Timeliness; PCQ = Personal contact quality; OA = Order accuracy; OC = Order condition; ODH = Order discrepancy handling; CR = Convenience of return; CT = Customer trust; CS = Customer satisfaction; RI = Repurchase intention; EW = Electronic word-of-mouth.

In summary, the evaluation of both convergent and discriminant validity demonstrated satisfactory results, indicating that the constructs were well-designed and robustly operationalized. These findings confirm that the measurement model is reliable and valid, providing a solid foundation for proceeding with hypothesis testing and ensuring the accuracy and credibility of the study’s conclusions.

#### 4.1.3. *Multicollinearity assessment.*

The Variance Inflation Factor (VIF) was used to identify potential linear relationships or multicollinearity among independent variables in multiple linear regression models [191]. Following the recommendation of [181], VIF values should remain below 3 (VIF < 3). In this study, VIF values ranged from 1.268 to 1.830 (Table 1), indicating that multicollinearity was not present. These results further reinforce the validity and reliability of the measurement model.

In conclusion, the measurement model demonstrated strong reliability, convergent validity, and discriminant validity, with no evidence of multicollinearity. These results establish a solid foundation for the subsequent structural model assessment and hypothesis testing.

### 4.2. *Structural model assessment*

#### 4.2.1. *Hypothesis testing.*

**Fig 2:**
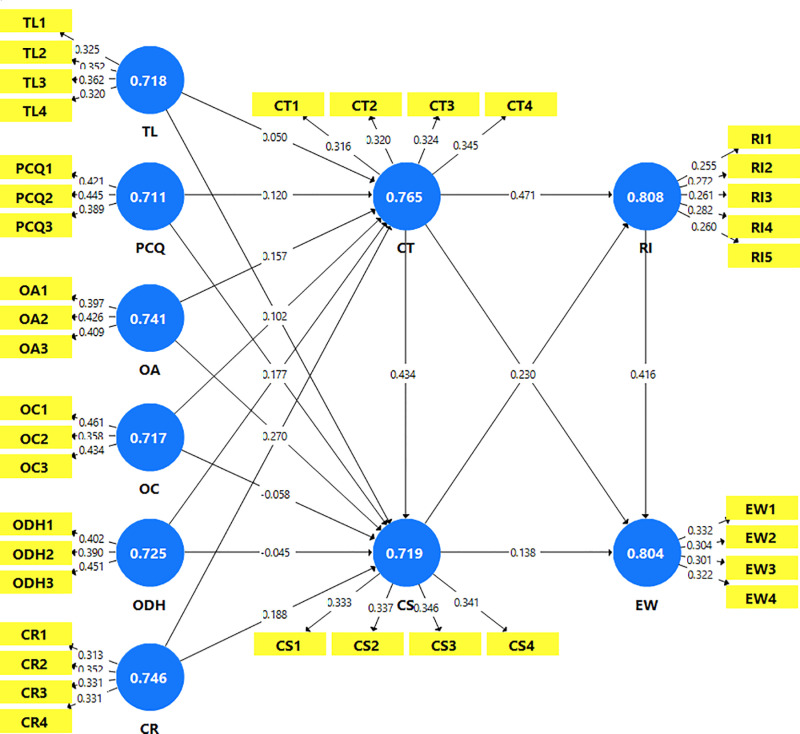
Results of PLS-SEM Analysis.

Hypothesis testing was conducted using the Partial Least Squares Structural Equation Modeling (PLS-SEM) approach, enhanced by bootstrapping techniques. To ensure robust and reliable results, the bootstrapping procedure utilized 1,000 resampled iterations with replacement, as recommended by Sarstedt et al. [192], providing a rigorous method for assessing the statistical significance of the model’s path coefficients. The findings from the hypothesis testing, as detailed in Table 6 and illustrated in Figure 2, provide critical insights into the interrelationships among constructs within the research model, shedding light on the dynamics of customer trust, satisfaction, and behavioral intentions. These findings indicate that most of the proposed hypotheses were supported, while H1, H6, H8, and H10 were not statistically significant. Specifically, the analysis revealed that timeliness (TL) did not exert a statistically significant impact on customer trust (CT) (H1: β = 0.05, p = 0.360), indicating that while customers appreciate timely service, it does not independently establish trust. This finding suggests that punctuality alone is perceived as a basic expectation rather than a trust-building factor. In addition, timeliness (TL) had the weakest impact on customer satisfaction (CS) among the tested paths, with H2 showing the smallest coefficient (β = 0.102, p = 0.039). This indicates that while punctual delivery does contribute to satisfaction by meeting baseline expectations and reducing customer inconvenience, its influence is relatively limited compared to other factors. Timeliness fulfills fundamental service standards, but building stronger satisfaction—and especially trust—requires more than just on-time delivery; it demands consistent, high-quality service across the entire customer experience.

**Table 6:** Hypothesis testing results.

Hypothesis	Paths	Coefficient (β)	Sample Mean (M)	Standard Deviation (S.D)	T Statistic	P Values	f² (Effect Size)	Results
**H1**	TL - > CT	0.050	0.053	0.055	0.916	0.360	0.003	Rejected
**H2**	TL - > CS	0.102	0.102	0.049	2.066	0.039	0.011	Accepted
**H3**	PCQ - > CT	0.120	0.124	0.060	2.016	0.044	0.014	Accepted
**H4**	PCQ - > CS	0.155	0.152	0.063	2.471	0.014	0.021	Accepted
**H5**	OA - > CT	0.157	0.152	0.053	2.958	0.003	0.027	Accepted
**H6**	OA - > CS	0.025	0.023	0.052	0.477	0.633	0.001	Rejected
**H7**	OC - > CT	0.134	0.132	0.059	2.296	0.022	0.022	Accepted
**H8**	OC - > CS	-0.058	-0.055	0.051	1.123	0.262	0.004	Rejected
**H9**	ODH - > CT	0.177	0.178	0.055	3.239	0.001	0.034	Accepted
**H10**	ODH - > CS	-0.045	-0.043	0.061	0.740	0.460	0.002	Rejected
**H11**	CR - > CT	0.270	0.272	0.064	4.202	0.000	0.070	Accepted
**H12**	CR - > CS	0.188	0.191	0.070	2.679	0.008	0.029	Accepted
**H13**	CT - > CS	0.434	0.433	0.065	6.636	0.000	0.176	Accepted
**H14**	CT - > RI	0.471	0.470	0.072	6.581	0.000	0.219	Accepted
**H15**	CT - > EW	0.285	0.285	0.060	4.781	0.000	0.082	Accepted
**H16**	CS - > RI	0.230	0.234	0.071	3.243	0.001	0.052	Accepted
**H17**	CS - > EW	0.138	0.142	0.050	2.775	0.006	0.023	Accepted
**H18**	RI - > EW	0.416	0.415	0.063	6.599	0.000	0.217	Accepted

*Notes* TL = Timeliness; PCQ = Personal contact quality; OA = Order accuracy; OC = Order condition; ODH = Order discrepancy handling; CR = Convenience of return; CT = Customer trust; CS = Customer satisfaction; RI = Repurchase intention; EW = Electronic word-of-mouth.

Personal contact quality (PCQ), on the other hand, emerged as a significant predictor of both CT (H3: β = 0.120, p = 0.044) and CS (H4: β = 0.155, p = 0.014), emphasizing that personalized and responsive service interactions enhance both trust and satisfaction. Expressing empathy and attentiveness builds confidence in the service provider and contributes to a more engaging customer experience. Similarly, order accuracy (OA) significantly impacted CT (H5: β = 0.157, p = 0.003), indicating that customers perceive accurate order fulfillment as a reflection of the retailer’s competence and commitment to meeting expectations, which fosters trust. However, OA did not significantly influence CS (H6: β = 0.025, p = 0.633), suggesting that while preventing errors is essential to avoid dissatisfaction, accurate delivery alone does not elevate satisfaction beyond the basic expectation of receiving the correct product. This indicates that customers may view order accuracy as a fundamental requirement rather than a differentiator, pointing out the need for retailers to complement accuracy with additional value-added services to enhance satisfaction.

Order condition (OC) significantly affected CT (H7: β = 0.134, p = 0.022), indicating that well-preserved and carefully handled orders enhance customer confidence, as they reflect the retailer’s commitment to quality and meticulous service. Conversely, OC did not significantly influence CS (H8: β = -0.058, p = 0.262), implying that while maintaining product condition is necessary to meet basic expectations, it does not actively enhance satisfaction. Customers may perceive receiving items in good condition as a fundamental aspect of the shopping experience, rather than as a factor that differentiates superior service quality. Effective handling of order discrepancies (ODH) also significantly influenced CT (H9: β = 0.177, p < 0.001), highlighting the critical role of problem resolution in fostering a sense of reliability and accountability. This implies that when retailers promptly address and rectify issues, customers are more likely to view the retailer as trustworthy. However, ODH did not significantly impact CS (H10: β = -0.045, p = 0.460), suggesting that while effective issue resolution prevents dissatisfaction, it does not actively enhance satisfaction. Customers may view problem-solving efforts as a necessary response rather than a proactive aspect of high-quality service, thus perceiving it more as damage control than a positive service attribute. Meanwhile, the convenience of return (CR) significantly affected both CT (H11: β = 0.270, p = 0.008) and CS (H12: β = 0.188, p < 0.001). This finding underscores the critical role of hassle-free return procedures in building customer confidence and satisfaction. Ensuring that customers feel assured about the ease of returning products, it reduces perceived risks associated with online purchases, thereby enhancing their overall trust. Additionally, an efficient and straightforward return process positively influences the post-purchase experience, making customers feel valued and supported, which in turn strengthens satisfaction.

The mediating roles of trust and satisfaction were also evident in the study. Among the tested relationships, trust (CT) showed the strongest direct impact on repurchase intention (RI), with H14 yielding the highest path coefficient (β = 0.471, p < 0.001). This underscores trust as the most powerful driver of repeat purchasing behavior. Trust also significantly influenced customer satisfaction (H13: β = 0.434, p < 0.001) and electronic word-of-mouth (EW) (H15: β = 0.285, p < 0.001). These results highlight the centrality of trust as a cornerstone of customer loyalty and advocacy, indicating that when customers trust a retailer, they are not only more satisfied but also more likely to make repeat purchases and share positive feedback. Strengthening and maintaining trust enhances customer satisfaction, loyalty, and fosters positive word-of-mouth, which are crucial for long-term success in the competitive e-commerce landscape. Additionally, CS had a significant impact on RI (H16: β = 0.230, p = 0.001) and EW (H17: β = 0.138, p = 0.006), indicating that satisfied customers are more likely to make repeat purchases and share favorable reviews. This finding emphasizes that enhancing customer satisfaction not only drives loyalty but also contributes positively to the retailer’s reputation, highlighting the dual role of satisfaction in both retention and advocacy. Finally, the significant effect of RI on EW (H18: β = 0.416, p < 0.001) indicates that customers who are inclined to make repeat purchases are also more likely to promote the brand through positive recommendations. This demonstrates that fostering strong repurchase intentions not only secures customer retention but also enhances brand advocacy, amplifying the retailer’s visibility and reputation. These findings collectively provide a comprehensive understanding of how LSQ dimensions influence customer perceptions and behaviors. They also emphasize the interconnected roles of trust and satisfaction in shaping repurchase intentions and eWOM, offering valuable insights for practitioners aiming to enhance customer loyalty in Vietnam’s dynamic e-commerce market.

Fig 2 illustrates the results of the PLS-SEM analysis, presenting the structural model with standardized path coefficients and outer loadings. The diagram highlights the relationships among key latent variables—such as logistics service quality (LSQ), trust, satisfaction, repurchase intention, and eWOM—alongside their associated measurement items. The strength and significance of each path are visually represented, indicating which hypotheses were supported. Reliability and validity indicators (e.g., outer loadings > 0.7) demonstrate the robustness of the model’s constructs and confirm the overall fit of the measurement and structural components.

#### 4.2.2. *Predictive power and relevance.*

The predictive power and relevance of the structural model were assessed using R-squared (R²), adjusted R-squared, and Q-squared (Q²) indices, providing insights into both in-sample explanatory power and out-of-sample predictive capability.

R-squared (R²) and adjusted R-squared indices were applied to evaluate the extent to which one or more independent variables explained variance in the dependent variables [193]. R² values indicate the explanatory power of the model, where higher values signify stronger predictive capability. R² values range from 0 to 1, with higher values indicating stronger explanatory power. While no universal threshold exists for acceptable R² values, Hair et al. [181] and Henseler et al. [186] classify R² values as substantial (≥ 0.75), moderate (≥ 0.50), or weak (≥ 0.25). As shown in Table 7, the R² values ranged from 0.416 to 0.534, indicating moderate explanatory power for the dependent variables. Adjusted R² values, which account for the number of predictors in the model and provide a more accurate reflection of explanatory power, ranged from 0.413 to 0.531. These results confirm that the structural model possesses adequate in-sample predictive capability, sufficiently explaining the variance in the dependent constructs.

**Table 7:** R² and Q² results.

	R Square (R²)	R Square Adjusted	Q Square (Q²)
**CS**	0.489	0.482	0.256
**CT**	0.524	0.518	0.299
**EW**	0.534	0.531	0.331
**RI**	0.416	0.413	0.231

*Notes* CT = Customer trust; CS = Customer satisfaction; RI = Repurchase intention; EW = Electronic word-of-mouth.

To assess the out-of-sample predictive capability and the overall quality of the structural model, the Q-squared (Q²) index was used. Q² serves as an indicator of the overall quality of the component models within the structural model [194]. A Q² value greater than 0 signifies that the structural model has predictive relevance [181,186]. Using the Blindfolding technique with an omission distance of 7, the analysis yielded Q² values ranging from 0.231 to 0.331 (Table 7). These results indicate that the model demonstrates strong overall quality and satisfactory predictive accuracy. Specifically, Q² values above 0 confirm that the endogenous constructs within the model have sufficient explanatory and predictive relevance.

In summary, the structural model exhibits moderate explanatory power (based on R² and adjusted R² values) and good predictive accuracy (based on Q² values), underscoring its robustness and reliability in capturing the relationships among the constructs under investigation. These findings provide confidence in the model’s ability to predict outcomes both within and beyond the sample data.

#### 4.2.3. *Effect size (f²).*

The effect size (f²) is a crucial measure used to evaluate the relative impact of each independent variable within a structural model, providing deeper insights beyond the overall explanatory power indicated by R² [195]. While R² measures the combined variance explained by all predictors, f² assesses the unique contribution of each predictor to determine whether its inclusion significantly enhances the model’s explanatory capability [196]. In the context of structural equation modeling, f² values of 0.02, 0.15, and 0.35 are interpreted as small, medium, and large effect sizes, respectively [186].

As shown in Table 6, the f² values obtained in this study range from 0.001 to 0.219, indicating a diverse range of effect sizes across the model’s paths. The most substantial effects are observed for the CT to RI path (0.219) and the RI to EW path (0.217), indicating that CT significantly impacts RI, and RI significantly influences EW. These high f² values suggest that removing these constructs would notably reduce the model’s explanatory power. Conversely, paths such as TL to CT (0.003) and OA to CS (0.001) show negligible effects, indicating minimal contributions. Medium effect sizes, like the CT to CS path (0.176), demonstrate moderate influence, while smaller effect sizes, such as the CS to EW path (0.023), indicate weaker but relevant associations.

The findings demonstrate the diverse impact of predictor variables within the structural model, with effect sizes ranging from negligible to substantial. These results validate the model’s ability to accurately capture the relationships among key constructs, offering valuable insights for both theoretical understanding and practical application.

## 5. Discussion

The findings of this study provide valuable insights into how logistics service quality (LSQ) influences the behavioral intentions of Generation Z consumers in Vietnam’s rapidly evolving e-commerce industry. It underscores the importance of specific LSQ dimensions in shaping trust, satisfaction, and behavioral outcomes such as repurchase intention and electronic word-of-mouth (eWOM). The findings not only validate but also expand upon existing literature by highlighting the unique characteristics of Generation Z and the Vietnamese e-commerce landscape.

The study revealed that timeliness did not significantly influence customer trust (H1 rejected), although it had a significant positive effect on satisfaction (H2 accepted). This suggests that while prompt delivery may enhance satisfaction, it is insufficient as a standalone factor to establish trust among Generation Z consumers. This demographic likely perceives timeliness as a fundamental expectation rather than a differentiator, reflecting their higher service standards in a digitally driven era. This could be due to their reliance on real-time tracking and proactive communication mechanisms, which help alleviate concerns about late deliveries and shift trust formation toward other service dimensions [77]. This finding diverges from previous studies, such as those by Do et al. [5] and Rebollo and Hinlayagan [197], which consistently highlighted timeliness as a critical determinant of trust. These studies emphasized that timely delivery symbolizes reliability and a commitment to fulfilling service promises, particularly in regions where customers place high value on punctuality. In Vietnam, where the e-commerce sector is intensely competitive, prompt delivery plays a crucial role in fostering trust and satisfaction [198]. However, this study aligns with insights from Kalia et al. [199], who argued that trust is often built through consistent performance across multiple service dimensions rather than reliance on a single feature like prompt delivery. One possible explanation for this weaker role of timeliness in influencing trust is the growing use of artificial intelligence (AI) and predictive analytics in logistics, which provide customers with real-time updates and estimated delivery times, thereby reducing uncertainty and the need to rely solely on punctuality as an indicator of reliability [200]. For businesses, this finding underscores the importance of integrating timeliness with broader trust-building strategies. Transparent communication about delivery timelines, proactive issue resolution, and reliable after-sales service are critical components for fostering trust. By offering a holistic approach that exceeds baseline expectations, businesses can differentiate themselves in a competitive market and cultivate stronger customer relationships.

The findings highlight the significance of personal contact quality (PCQ) as a driver of both trust (H3 accepted) and satisfaction (H4 accepted). This aligns with traditional service quality frameworks that emphasize the critical role of interpersonal interactions in building trust and ensuring customer satisfaction. However, the pronounced impact of PCQ in this study reflects the evolving expectations of Generation Z, a demographic that values high-quality personal interactions, particularly for issue resolution and customization. While they appreciate these human elements, Generation Z also demands seamless integration with digital solutions. According to Kim et al. [201], this demographic views technology-driven efficiency and human support as complementary rather than mutually exclusive. This dual preference is further reinforced by Maj [202], who found that Generation Z’s comfort with digital tools enhances their trust in e-commerce platforms when such tools are combined with competent and personalized service. Consequently, e-commerce platforms must prioritize delivering exceptional personal interactions while integrating advanced digital tools to optimize both trust and satisfaction.

In contrast, order accuracy positively influenced trust (H5 accepted) but did not significantly impact satisfaction (H6 rejected), challenging conventional logistics service quality (LSQ) assumptions. Traditionally, order accuracy has been considered a key determinant of satisfaction [203]. However, this study suggests a shift in perception among Generation Z consumers, who regard order accuracy as a baseline expectation rather than a distinguishing factor. This perspective stems from the increasing sophistication of logistics technologies, which have normalized accurate deliveries and raised the bar for what defines a satisfying e-commerce experience [204]. Businesses must therefore treat order accuracy as a non-negotiable standard while focusing on enhancing other service dimensions to create a differentiated and delightful customer experience. Moreover, while the absence of order accuracy can result in dissatisfaction, its presence alone does not actively enhance satisfaction but plays a critical role in building trust [88]. This finding underscores the need for e-commerce businesses to consistently meet fundamental requirements like accuracy while exploring innovative ways to exceed customer expectations. For example, adding personalized touches, offering tailored recommendations, or providing exceptional after-sales support can elevate the overall consumer experience, thereby fostering stronger trust and satisfaction. This unexpected finding that order accuracy builds trust but not satisfaction challenges established service quality theory. It suggests Generation Z views accuracy as a trust-building baseline rather than a satisfaction driver, possibly due to their extensive exposure to precise e-commerce systems from major platforms [205]. This indicates a potential shift in how digital natives evaluate service quality, where technical excellence maintains trust but fails to generate emotional satisfaction. The finding also suggests that e-commerce platforms may need to rethink their approach to satisfaction, focusing on elements beyond mere accuracy such as personalized experiences or social shopping features that resonate with Generation Z’s distinct preferences.

Additionally, the study discovers that the condition of delivered orders significantly influenced trust (H7 accepted) but had no significant impact on satisfaction (H8 rejected). This result contrasts with prior research, such as Duarte et al. [206], which identified order condition as a driver of satisfaction. While intact packaging and product quality are undoubtedly essential, Generation Z appears to view these attributes as fundamental requirements rather than contributors to satisfaction [207]. This finding reflects a generational shift in service quality perceptions, driven by the consistent performance of leading e-commerce platforms. Over time, attributes like proper packaging and undamaged products have become integral to the e-commerce experience, no longer seen as differentiators but as standard requirements [57]. Generation Z appears to distinguish between core service dimensions, which ensure trust through reliability, and value-added features, which are needed to evoke satisfaction and loyalty. While meeting basic requirements establishes trust, it does little to exceed expectations or create delight. To enhance satisfaction among Generation Z consumers, businesses need to go beyond meeting these fundamental requirements. Innovations such as sustainable and eco-friendly packaging, aesthetically pleasing designs, or interactive packaging elements can align with this demographic’s values and preferences. The surprising disconnect between order condition’s impact on trust versus satisfaction may stem from Generation Z’s unique values and experiences. Their digital-first mindset and environmental consciousness could mean traditional protective packaging, while trust-building creates insufficient or even negative emotional responses [208]. This challenges conventional assumptions about physical service evidence driving satisfaction. Moreover, in an era where unboxing experiences are frequently shared on social media, standard packaging might fall short of creating the memorable experiences that Generation Z associates with satisfaction [209]. This suggests the need for innovative, sustainable packaging solutions that align with both functional and emotional expectations.

Similarly, order discrepancy handling played a critical role in shaping trust (H9 accepted) but had no significant impact on satisfaction (H10 rejected). This outcome may be attributed to the proactive, technology-driven practices employed by Vietnamese e-commerce firms, which minimize consumer effort in resolving issues. For example, many platforms now provide mobile apps with features like real-time tracking and one-click resolution of discrepancies, setting a high benchmark for service recovery [210]. These tools have normalized efficient problem resolution, making it an expected feature rather than an exceptional service experience. The finding underscores a key insight: seamless service recovery acts more as a trust-builder than a satisfaction enhancer. For Generation Z, smooth resolution processes are essential but insufficient to generate satisfaction. This aligns with research by Xiaohui et al. [211], which emphasizes the role of effective post-purchase services in establishing long-term trust and customer loyalty. The finding that effective problem resolution builds trust but not satisfaction contradicts service recovery paradox theory. This may reflect Generation Z’s high baseline expectations from automated resolution systems or suggest that the emotional impact of problems persists despite resolution. Modern e-commerce platforms’ sophisticated complaint handling systems may have normalized efficient resolution, making it a trust requirement rather than a satisfaction booster [212]. This indicates traditional service recovery metrics may need recalibration for digital-native consumers who expect seamless, immediate problem resolution. The challenge for e-commerce platforms lies in finding ways to transform standard problem resolution into satisfaction-generating experiences.

Moreover, the results show that return convenience is a critical determinant of both trust and satisfaction (H11 and H12 accepted), emphasizing the importance of a hassle-free return process in reducing perceived risks and enhancing the overall customer experience. As noted by Thu et al. [213], flexible and customer-friendly return policies are particularly vital for retaining Generation Z consumers, who prioritize convenience and transparency in e-commerce transactions. In a competitive market like Vietnam’s e-commerce sector, businesses that offer seamless returns can stand out by improving customer satisfaction and fostering loyalty. The quality of post-purchase support, including the ease of returns, plays a crucial role in customer retention, suggesting that companies that excel in this area will be better positioned to thrive.

The study also confirmed the strong relationships between trust, satisfaction, repurchase intention, and eWOM, reinforcing the trust-satisfaction-loyalty chain in the Vietnamese e-commerce context. Trust was found to be a key driver of satisfaction (H13 accepted), repurchase intention (H14 accepted), and eWOM (H15 accepted), aligning with findings by Mittal et al. [214], who assert that trust forms the foundation of positive customer behaviors. Similarly, satisfaction was shown to have a significant influence on both repurchase intention (H16 accepted) and eWOM (H17 accepted), supporting the work of Ruiz-Alba et al. [215] and Salam et al. [216], who note that satisfied Generation Z customers are more likely to return and recommend brands through social media. Additionally, the significant relationship between repurchase intention and eWOM (H18 accepted) underscores how loyalty and advocacy behaviors are interconnected. Loyal customers, through word-of-mouth and social media, can significantly boost brand visibility and attract new customers.

This study makes a substantial contribution to the understanding of service quality (LSQ) in Vietnam’s e-commerce industry, particularly in relation to Generation Z. The findings suggest that while certain traditional service quality dimensions remain essential, others need to be rethought to align with the evolving preferences of this demographic. For businesses, the practical implications are clear: focusing on digital-first experiences, optimizing logistics systems for speed and accuracy, and delivering outstanding post-purchase services are key strategies. These efforts will be instrumental in building trust, satisfaction, and loyalty among Generation Z consumers, ensuring long-term success in the highly competitive and rapidly evolving Vietnamese e-commerce landscape.

## 6. Implications

The findings of this study hold significant theoretical and practical implications, particularly for understanding logistics service quality (LSQ) in the context of Generation Z consumers and e-commerce. By bridging academic insights with actionable strategies, this research contributes to advancing both scholarly knowledge and industry practices.

### 6.1. Theoretical implications

This study enriches LSQ literature by challenging and expanding traditional frameworks, particularly in the context of Generation Z’s evolving expectations. The diminished role of timeliness in fostering trust among Generation Z consumers underscores a generational shift, where baseline expectations for speed and reliability necessitate a reevaluation of LSQ dimensions. Unlike earlier assumptions emphasizing timeliness as a trust driver, the study reveals that Generation Z values it as a standard feature, highlighting the need for supplementary trust-building elements like transparency and reliability in other service areas.

Additionally, the study enriches the understanding of the trust-satisfaction-loyalty chain by demonstrating the nuanced effects of individual LSQ dimensions. While attributes like personal contact quality and return convenience emerge as dual influencers of trust and satisfaction, elements such as order accuracy and order condition primarily build trust rather than satisfaction. These findings emphasize the need to differentiate between the roles of LSQ components in digital environments, marking a departure from traditional offline service quality models. Furthermore, the study advances post-purchase service quality literature by establishing the pivotal role of order discrepancy handling and return convenience in building trust and satisfaction. This insight contributes to service recovery theory by highlighting the heightened expectations of Generation Z for seamless problem resolution and efficient service recovery in e-commerce. Such findings offer a theoretical foundation for exploring the evolving nature of consumer expectations in a rapidly digitalizing marketplace.

### 6.2. Practical implications

From a practical standpoint, this research offers valuable insights for e-commerce operators and logistics service providers in emerging markets. The findings emphasize the critical importance of delivery timeliness, urging companies to invest in advanced logistics infrastructure and last-mile delivery solutions to meet the demand for speed and reliability. Moreover, the significant impact of personal contact quality on both trust and satisfaction underscores the importance of maintaining high standards in personal interactions while complementing them with advanced technological tools. Businesses should invest in training staff to deliver empathetic and competent support, particularly for complex issues, while leveraging digital solutions such as AI chatbots and real-time assistance to streamline routine queries and interactions. These efforts should align with Generation Z’s preference for technology-driven solutions, enhancing trust through efficient, user-friendly digital experiences. To implement these digital solutions effectively, companies should focus on developing comprehensive delivery management systems. This includes AI-powered delivery time prediction with precise delivery windows, coupled with mobile apps offering visual package tracking and real-time driver location updates. Customer experience can be enhanced through personalized delivery profiles storing individual preferences and delivery instructions. For customer service automation, companies should deploy intelligent chatbots trained on company-specific logistics data for swift query resolution. A systematic notification system should keep customers informed throughout key delivery touchpoints, from order confirmation to final delivery. In terms of order fulfillment, maintaining high standards of accuracy and product condition is essential to meet baseline expectations. However, companies must go beyond these fundamentals by incorporating value-adding features such as sustainable packaging, personalized delivery options, and real-time tracking capabilities to foster customer satisfaction and differentiate themselves in a competitive market.

The research also highlights the importance of robust post-purchase services in building customer trust and satisfaction. This suggests that businesses should invest in automated return systems, accessible drop-off locations, and swift refund processing to streamline return convenience and strengthen service recovery capabilities. Companies can revolutionize their returns process through an integrated digital returns management system. This should feature QR code-based return initiation for instant label generation, complemented by partnerships with ride-hailing services for on-demand pickup. A multi-channel return network incorporating doorstep collection, convenience store drop-offs, and smart lockers provides customers with flexible options. The refund process should be automated and expedited, supported by a digital tracking portal and integration with popular e-wallets for quick refunds. Effective resolution of order discrepancies, as highlighted in this study, is particularly crucial for Generation Z consumers who expect seamless and immediate solutions to service issues. Moreover, the strong relationship between trust, satisfaction, repurchase intention, and eWOM underscores the importance of fostering trust through transparent communication and reliable service delivery. Companies should also leverage satisfied customers as brand advocates by encouraging positive social media sharing. To leverage social proof effectively, companies should implement a comprehensive social engagement strategy. This includes developing a gamified review system that rewards detailed feedback and visual content sharing. Social media integration should feature easy sharing options with customizable templates and branded hashtags. Strategic post-purchase surveys and a structured referral program can drive organic growth, while social media-friendly packaging with interactive elements encourages user-generated content that amplifies brand presence across digital platforms. This strategy not only amplifies eWOM but also enhances brand visibility and customer acquisition in the digital age.

For industry stakeholders in Vietnam and similar emerging markets, these findings provide a roadmap for service quality optimization targeting Generation Z consumers. Success requires balancing operational excellence in core service areas with innovation that appeals to Generation Z’s unique preferences. Companies should prioritize digital transformation initiatives while maintaining focus on fundamental service quality dimensions that drive customer loyalty and positive word-of-mouth in the competitive e-commerce landscape.

## 7. Limitations and recommendations

While this study provides valuable insights into the influence of logistics service quality (LSQ) on Generation Z consumers in Vietnam’s e-commerce sector, certain limitations should be acknowledged. Addressing these limitations not only strengthens the interpretation of the findings but also offers pathways for future research to deepen understanding of LSQ in a rapidly evolving e-commerce landscape.

First, the cross-sectional nature of the study limits its ability to capture the dynamic and evolving nature of customer perceptions and behaviors over time. As Generation Z consumers gain more experience with e-commerce platforms or as logistics services evolve, their expectations and evaluations of LSQ dimensions may shift. Future research employing a longitudinal design could offer richer insights into how trust and satisfaction are built and maintained over time, providing a clearer picture of temporal changes in consumer attitudes and behavior. Second, the study’s focus on Vietnam, while providing valuable insights into an important emerging market, may limit the generalizability of findings to other cultural and economic contexts. Vietnam’s unique consumer culture and digital transformation trajectory may differ significantly from those of more mature or less developed e-commerce markets. To validate and expand upon these findings, replication studies in diverse geographic and cultural settings are recommended. Such comparative analyses could uncover universal versus context-specific aspects of LSQ and its impact on Generation Z consumers. Third, the reliance on quantitative methods, while ensuring statistical rigor, may not fully capture the depth and complexity of Generation Z’s decision-making processes and emotional responses to LSQ. The addition of qualitative approaches, such as in-depth interviews or focus groups, would provide richer, more nuanced insights into the motivations and expectations that drive their perceptions of logistics services. This mixed-methods approach could also illuminate the emotional and experiential aspects of LSQ, which are often difficult to quantify.

Moreover, while the study explored key LSQ dimensions, it may not have comprehensively captured all relevant aspects of contemporary e-commerce logistics services. Emerging factors such as sustainable delivery options, advanced tracking technologies, or innovative last-mile solutions may significantly influence customer behavior but were not addressed in this research. Future studies should integrate these and other evolving dimensions of LSQ to provide a more holistic understanding of their impact on trust, satisfaction, and loyalty. Finally, the exclusive focus on Generation Z consumers may overlook generational differences that could contextualize these findings. Conducting comparative studies that include other age cohorts, such as Millennials or Baby Boomers, would provide valuable insights into how LSQ expectations and their behavioral impacts vary across generations. Such research would also help identify whether the preferences of Generation Z are distinct or part of broader trends in consumer behavior. In summary, these limitations provide opportunities for future research to build upon this study’s findings and further enhance our understanding of LSQ in the evolving e-commerce landscape.

## 8. Conclusion

This study provides a thorough examination of the interplay between logistics service quality (LSQ) dimensions and the behavior of Generation Z consumers within Vietnam’s dynamic e-commerce landscape. Specifically, it aims to explore how specific LSQ factors—such as timeliness, personal contact quality, order accuracy, order condition, order discrepancy handling, and return convenience—impact trust and satisfaction which in turn driving repurchase intention, and electronic word-of-mouth (eWOM) among this digitally native demographic. Guided by the research objectives, a quantitative approach was employed to collect data from 495 Generation Z participants with prior experience in online shopping through e-commerce platforms. The research model and hypotheses were rigorously analyzed using Partial Least Squares Structural Equation Modeling (PLS-SEM), yielding insights that advance both theoretical understanding and practical strategies in e-commerce logistics.

The findings of this study highlight the need to re-evaluate traditional logistics service quality (LSQ) frameworks to align with the evolving expectations of digitally native consumers, particularly Generation Z. Among the LSQ dimensions examined, personal contact quality emerged as a significant driver of both trust and satisfaction. This underscores the enduring importance of human interaction in fostering positive customer perceptions, even within a digitally dominated marketplace. However, dimensions such as order accuracy and order condition influenced trust but did not significantly impact satisfaction. These results suggest that Generation Z perceives these attributes as basic expectations rather than features that differentiate service providers. Moreover, timeliness demonstrated a strong positive effect on satisfaction but did not significantly impact trust, indicating that prompt delivery alone does not suffice to establish credibility. Conversely, post-purchase services, such as return convenience and discrepancy handling, were confirmed as critical for building trust and satisfaction, reflecting the heightened expectations of Generation Z for seamless, technology-driven service recovery. The study further confirmed the strong interrelationships among trust, satisfaction, repurchase intention, and electronic word-of-mouth (eWOM). Trust emerged as a pivotal factor driving satisfaction, which in turn significantly influenced both repurchase intentions and positive eWOM behaviors. This dynamic underscores the critical role of trust and satisfaction in fostering customer loyalty and advocacy, with satisfied and loyal customers amplifying brand visibility through social media and other online channels.

Theoretically, this research makes significant contributions by expanding the understanding of LSQ in the context of emerging markets and younger consumer demographics. It challenges conventional service quality paradigms and offers a revised framework that incorporates the shifting preferences of digital natives. The findings also provide a foundation for further exploration, particularly in investigating emerging LSQ dimensions, conducting cross-generational comparisons, and exploring the evolving dynamics of e-commerce in diverse cultural settings. By bridging generational and regional gaps in LSQ literature, this study offers a more holistic perspective on how digital transformation influences logistics service expectations across varying market conditions. Furthermore, this research provides an empirical foundation for future studies to explore comparative analyses between Generation Z consumers in developed and emerging markets, contributing to a broader understanding of global e-commerce logistics trends.

From a practical perspective, this research provides actionable recommendations for e-commerce stakeholders aiming to cater to Generation Z consumers. Businesses should prioritize investments in efficient logistics infrastructure, advanced digital interfaces, and robust post-purchase support mechanisms, such as automated return systems and transparent communication. These measures align with Generation Z’s preference for self-service technologies over traditional customer interaction. By addressing these priorities, e-commerce operators in Vietnam and similar emerging markets can enhance their competitive edge and foster stronger customer loyalty in a rapidly evolving industry.

In conclusion, as Generation Z’s influence on the e-commerce sector continues to grow, businesses must adapt to their distinct expectations for service quality. This study not only addresses these challenges but also serves as a foundation for future research on LSQ, particularly in exploring new logistics innovations and technological advancements tailored to digital-native consumers. By bridging theoretical gaps and offering practical guidance, it contributes significantly to advancing the field of LSQ and its application in meeting the needs of a new generation of consumers in the digital age.

## Supporting information

S1 File Survey questionnaire. (PDF)

S2 File Dataset used in analysis. (XLSX)
