# Supplementary material for: Roles of logistics service quality in shaping generation Z customers’ repurchase intention and electronic word of mouth in E-commerce industry
Source: PLoS One. 2025 May 28;20(5):e0323962. doi: 10.1371/journal.pone.0323962 (PMC12118890; doi:10.1371/journal.pone.0323962)
Supplement: S1 File — (PDF) [file pone.0323962.s001.pdf]

QUESTIONNAIRE

“Roles of Logistics Service Quality in Shaping Generation Z Customers’ Repurchase Intention and Electronic Word of Mouth in E-Commerce Industry”

Dear participant,

We are conducting a study to examine the impact of logistics service quality (LSQ) on repurchase intention and electronic word-of-mouth (eWOM) behavior among Generation Z customers in Vietnam's e-commerce sector. This study focuses on how specific aspects of logistics services—such as timeliness, personal contact quality, order accuracy, order condition, order discrepancy handling, and return convenience—affect purchasing decisions and online reviews of Generation Z customers.

Your valuable insights will help us identify the key factors that drive repurchase intentions and encourage eWOM behavior, contributing to the improvement of e-commerce services in Vietnam.

Participation in this survey is entirely voluntary, and all the information you provide will remain confidential and used exclusively for academic research purposes. We kindly request a few minutes of your time to complete the survey.

Thank you for your contribution to this research.

Do you agree to participate in this survey and give consent for using your information for research purposes?

- Yes
- No

I. Screening Section

Have you ever shopped on e-commerce platforms? (e.g., TikTok, Shopee, Lazada, Tiki, etc.)

- Yes
- No

II. Demographics Section

2.1 Gender:

- Male
- Female
- Not to specify

2.2 Age:

- o 18 to 22 years old
- o 23 to 26 years old
- o 27 to 29 years old

2.3 What is your monthly income?

- o Less than 5 million VND
- o From 5 to 10 million VND
- o From 10 to 20 million VND
- o From 20 to 30 million VND
- o More than 30 million

2.4 What is your monthly spending on e-commerce platforms (VND)?

- o Less than 1 million VND
- o From 1 to 3 million VND
- o From 3 to 5 million VND
- o From 5 to 7 million VND
- o More than 7 million VND

2.5 What is your monthly shopping frequency on e-commerce platforms?

- o Less than once a month
- o 1 - 2 times per month
- o 3 - 5 times per month
- o More than 5 times per month

2.6 What is your preferred online shopping platform? *(Multiple options)*

- o TikTok
- o Shopee
- o Lazada
- o Tiki
- o Other:_____

III. Constructs and Items Section

The following questions pertain to your perceptions of logistics service quality in the e-commerce sector. Please indicate your level of agreement with the statements below by selecting a rating from 1 to 5.

1 = Strongly disagree

2 = Disagree

3 = Neutral

4 = Agree

5 = Strongly agree

**Timeliness** (Mentzer et al. (2001); Jiang et al. (2021); Akıl and Ungan (2022))	1	2	3	4	5

1) "The time between placing an online order and receiving the delivery is short."					
2) "The goods are delivered on the promised date."					
3) "If there's a delay, the logistics provider quickly reschedules the delivery."					
4) "The rate of non-compliance with set delivery times is very low."					
**Personal Contact Quality** (Bienstock et al. (2008); Lin et al. (2023))	1	2	3	4	5
1) "The logistics service employees possess adequate knowledge and experience to handle inquiries and problems competently."					
2) "The logistics service employees consistently display a positive attitude when addressing my concerns."					
3) "The logistics service employees maintain a courteous demeanor when dealing."					
**Order Accuracy (**Bienstock and Royne (2010); Akıl and Ungan (2022))	1	2	3	4	5
1) "Deliveries consistently contain the correct items."					
2) "The quantity of items delivered is always accurate."					
3) "Delivered products match the order specifications (e.g., model, color)."					
**Order Condition** (Akıl and Ungan (2022); Bienstock and Royne (2010))	1	2	3	4	5
1) "The product I ordered was delivered with appropriate protection."					
2) "Product is rarely damaged due to shipping method."					
3) "Product is rarely damaged due to handling by the shipping unit."					
**Order Discrepancy Handling (ODH)** (Mentzer et al. (2001); Akıl and Ungan (2022))	1	2	3	4	5
1) "It is easy to report order discrepancies to the seller."					
2) "The seller offers satisfactory solutions for order discrepancies."					

3) "Overall, the seller provides strong support in resolving product issues."					
**Convenience of Return** (Kim et al. (2014); Yang et al. (2020); Correa et al. (2021))	1	2	3	4	5
1) "I can return products for free when I shop online."					
2) "The return process is easy when I shop online."					
3) "I can return products within a specified time when I shop online."					
4) "I am able to return used products in some cases when I shop online."					
**Customer Trust** (Falahat et al. (2019))	1	2	3	4	5
1) "I believe the e-commerce logistics service is trustworthy."					
2) "I believe the e-commerce logistics service keeps promises and commitments."					
3) "I believe the e-commerce logistics service always has my best interests in mind."					
4) "I believe the e-commerce logistics service meets my expectations."					
**Customer Satisfaction** (Kim et al. (2014); Yang et al. (2020); Correa et al. (2021))	1	2	3	4	5
1) "I am satisfied with the e-commerce retailer's logistics services."					
2) "The logistics services for e-commerce purchases fulfill my demand."					
3) "The logistics experience I had with online retailers was exactly what I needed."					
4) "Using the logistics services offered by retailers on e-commerce platforms was the right one."					
**Repurchase Intention** (Hsu et al. (2015); Wijaya et al. (2018))	1	2	3	4	5
1) "I am highly likely to continue purchasing products from this e-retailer in the future."					

2) "I intend to continue shopping with this e-retailer rather than switch to others."					
3) "I will be back to repurchase products from this e-retailer in the future."					
4) "I prefer to buy products from this e-retailer rather than other online stores."					
5) "I will prioritize this e-retailer for future purchases."					
**Electronic Word-of-Mouth** (Mim et al. (2022))	1	2	3	4	5
1) "I will recommend this e-retailer to others through e-commerce platforms."					
2) "I will speak of the good sides of this e-retailer on social media."					
3) "I will be proud to say to others that I am a customer of this e-retailer."					
4) "I will speak favorably of this e-retailer to others."					

Thank you very much for your support!
